# Physiological and proteomic responses to salt stress in chloroplasts of diploid and tetraploid black locust (*Robinia pseudoacacia* L.)

**DOI:** 10.1038/srep23098

**Published:** 2016-03-15

**Authors:** Fanjuan Meng, Qiuxiang Luo, Qiuyu Wang, Xiuli Zhang, Zhenhua Qi, Fuling Xu, Xue Lei, Yuan Cao, Wah Soon Chow, Guangyu Sun

**Affiliations:** 1 College of Life Science, Northeast Forestry University, Harbin 150040, P.R. China; 2 Key Laboratory of Saline-Alkaline Vegetation Ecology Restoration in Oil Field (SAVER), Ministry of Education, Alkali Soil Natural Environmental Science Center, Northeast Forestry University, Harbin, 150040, P.R. China; 3 Division of Plant Science, Research School of Biology, The Australian National University, 46 Biology Place, Acton 2601, ACT, Australia

## Abstract

Salinity is an important abiotic stressor that negatively affects plant growth. In this study, we investigated the physiological and molecular mechanisms underlying moderate and high salt tolerance in diploid (2×) and tetraploid (4×) *Robinia pseudoacacia* L. Our results showed greater H_2_O_2_ accumulation and higher levels of important antioxidative enzymes and non-enzymatic antioxidants in 4× plants compared with 2× plants under salt stress. In addition, 4× leaves maintained a relatively intact structure compared to 2× leaves under a corresponding condition. NaCl treatment didn’t significantly affect the photosynthetic rate, stomatal conductance or leaf intercellular CO_2_ concentrations in 4× leaves. Moreover, proteins from control and salt treated 2× and 4× leaf chloroplast samples were extracted and separated by two-dimensional gel electrophoresis. A total of 61 spots in 2× (24) and 4× (27) leaves exhibited reproducible and significant changes under salt stress. In addition, 10 proteins overlapped between 2× and 4× plants under salt stress. These identified proteins were grouped into the following 7 functional categories: photosynthetic Calvin-Benson Cycle (26), photosynthetic electron transfer (7), regulation/defense (5), chaperone (3), energy and metabolism (12), redox homeostasis (1) and unknown function (8). This study provides important information of use in the improvement of salt tolerance in plants.

Polyploidy (chromosome doubling) is now widely viewed as a major force in plant evolution and diversification^1^. Polyploidy occurs in 70% of all angiosperms and is especially common in cultivated crops such as potato, cotton and wheat^2^. Most polyploids contain novel variations, which may contribute to speciation or the exploitation of eco-niches. Some polyploids are superior to their corresponding diploids in terms of tolerance to environmental stresses, such as drought^3^, heat^4^, nutrient-poor soils^1^ and salinity^5^. This increased tolerance may be attributable to duplicate gene expression or simply related to evolutionary time. To date, various morphological, physiological and molecular traits have been examined in polyploid plants. However, few studies have specifically tested the relationship between polyploidy and abiotic tolerance in woody plants. Thus, it is necessary to elucidate the precise mechanism responsible for stress tolerance in polyploid woody plants at the physiological and molecular level.

Salinity is one of the most important abiotic stressors that negatively affect plant growth and agricultural productivity. Generally, high salinity can disturb essential physiological processes by inducing water deficits, ion imbalance, hyperosmotic stress, nutritional imbalance, metabolic disorders and even death. To cope with salt stress, plants have evolved complex defense strategies. These include the up-regulation of antioxidant enzymes and antioxidants, energy metabolism modifications and the appearance or disappearance of some proteins. Although there is a clear understanding of how plants protect themselves from salt stress, the detailed mechanisms underlying tolerance in the chloroplasts of polyploid plants remain unclear.

In plants, the chloroplast is the organelle where biochemical and biophysical processes of photosynthesis occur^6^. Chloroplasts are more sensitive to salinity than other organelles. Reactive oxygen species (ROS) increase rapidly and excessively in chloroplasts, leading to the destruction of chloroplasts. This can be manifested as swelling of the thylakoids and a decrease in the extent of grana stacking in salt-treated potato plants^7^. High salinity also lowers stomatal conductance and the transpiration rate in leaves, leading to a decrease in photosynthesis^8^. To cope with salt stress, plants maintain higher activities of antioxidant enzymes in chloroplasts. For instance, Chinese cabbage increases its tolerance to salt stress after introducing maize cuprozinc-superoxide dismutase (Cu/Zn SOD) and/or Catalase (CAT) genes into its chloroplasts^9^. To date, physiological, ultrastructural and proteomic analyses have been used to detect changes in chloroplasts in response to high salinity in many plant species such as wheat^10^, maize^11^, Nicotiana benthamiana^12^ and rice^13^. However, knowledge regarding chloroplasts in woody species, especially polyploids, under salt stress conditions is still scarce.

Tetraploid black locust (*Robinia pseudoacacia* L.) is native to Korea and is a preferred tree species in the timber forest due to its rapid growth and good wood texture. Moreover, the fleshy leaves of this plant can be used as a fine feed for domestic fowl and livestock due to rich vitamin and mineral contents. Tetraploid black locust is a pioneer tree species due to its great adaptability to adverse conditions such as salt, drought, cold and pest infestation. Therefore, tetraploid black locust has high ecological and economic value. In this study, we investigated the response of chloroplasts in tetraploid black locust and its corresponding diploid in response to salt stress. We investigated (a) different responses in the chloroplasts of diploid and tetraploid black locust under salt stress at the physiological level and (b) how tetraploid black locust adjusted its chloroplast protein composition to enhance salt tolerance.

## Results

### Effect of Salt Treatment on Leaf Growth

#### Changes in enzymatic and non-enzymatic antioxidants and H_2_O_2_ content

**Figure 1:**
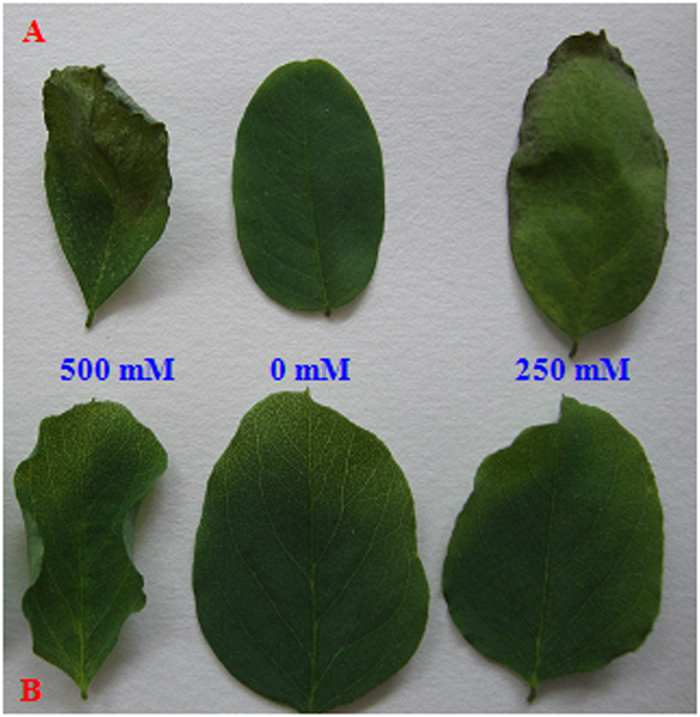
The morphological traits of 2× (**A**) and 4× (**B**) black locust leaves after 7 days of treatment under 0, 250, and 500 mM NaCl, respectively.

Diploid R. pseudoacacia (2×) leaves exhibited wilting and chlorosis. Many were etiolated from the leaf apex under 250 mM NaCl, as shown in Fig. 1. By contrast, tetraploid R. pseudoacacia (4×) leaves did not show any obvious etiolation under the same conditions (Fig. 1). In addition, 500 mM NaCl inhibited the growth of 2× leaves (Fig. 1); lighter damage was observed in 4× leaves under the same conditions (Fig. 1).

**Figure 2:**
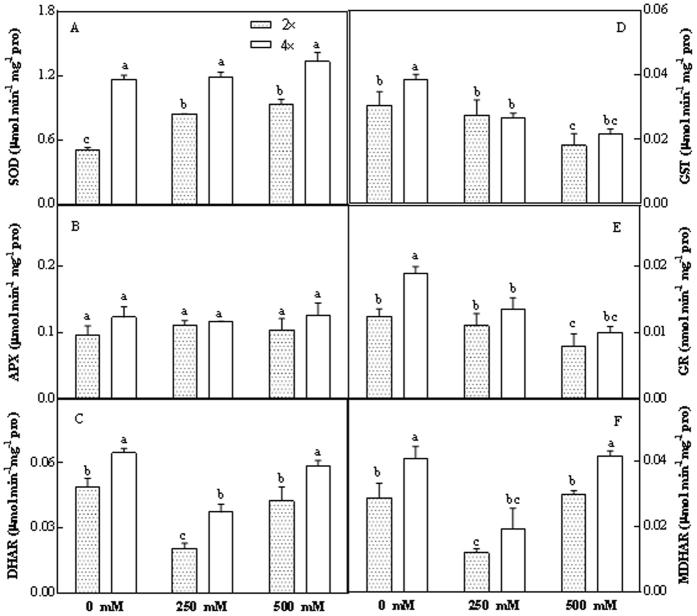
Changes in activities of SOD (**A**), APX (**B**), DHAR (**C**), GST (**D**), GR (**E**) and MDHAR (**F**) in chloroplasts of 2× and 4× leaves after salt treatment.

In some cases, enzymatic and non-enzymatic antioxidants were substantially affected by salt stress. This was observed in both 2× and 4× leaves. Superoxide dismutase (SOD. EC1.15.1.1) activity was marginally, though not significantly, higher in 4× leaves after salt treatment (Fig. 2A). Salt stressed 4× leaves showed marginally, though not significantly, higher ascorbate peroxidase (APX) activity compared to stressed 2× leaves (Fig. 2B). However, salt stress led to a significant decrease in Glutathione S-transferase (GST) and grana (GR); however, the activities of these components were higher in 4× leaves compared to 2× leaves (Fig. 2D,E). Dehydroascorbate reductase (DHAR, EC 1.8.5.1) and monodehydroascorbate reductase (MDHAR, EC 1. 6. 5. 4) activities decreased in the leaves of 2× and 4× plants treated with 250 mM NaCl treated relative to their controls; the activities of these enzymes increased in the 500 mM NaCl treated samples relative to samples treated with 250 mM NaCl. At any salt concentration, DHAR or MDHAR activity was significantly higher in 4× leaves compared to 2× leaves (Fig. 2C,F). Significant increases in AsA (ascorbic acid), (GSH) and H_2_O_2_ contents were found in both genotypes after salt treatment; contents were greater in 4× leaves compared to those in 2× leaves (Table 1).

#### Transmission electron microscopy observations

**Figure 3:**
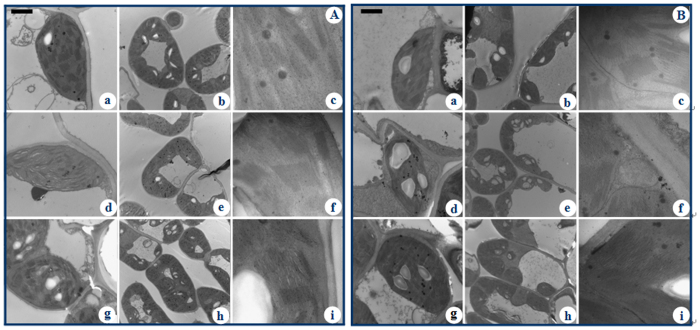
Ultrastructure of 2× (**A**) and 4× (**B**) black locust leaves after salt treatment. (a–c) Chloroplast under 0 mM NaCl; (d–f) Chloroplast under 250 mM NaCl; (g–i) Chloroplast under 500 mM NaCl. Bars in Figures (a,d,g) are 2 μm; Bars in Figures (b,e,h) are 5 μm; Bars in Figures (c,f,i) are 500 nm.

The ultrastructures of chloroplasts from both 2× and 4× leaves were normal under control conditions. Injuries were apparent in the chloroplasts of 2× leaves under salt stress (Fig. 3A,B). Under control conditions, the grana of both 2× and 4× leaves were well developed and highly stacked with normal thylakoids. In 2× leaves under treatment with 250 mM NaCl, marked swelling of grana and thylakoids with in incompact structures were observed. Under 500 mM NaCl treatment, the structure of thylakoids in 2× leaves was severely damaged. In addition, the number of grana severely decreased. There was a visible re-arrangement of chloroplasts, and their shape changed from a typical ellipsoidal shape to oval shaped (Fig. 3A). In contrast, the chloroplasts of 4× leaves maintained a relatively intact structure compared with those of 2× leaves under corresponding conditions (Fig. 3B).

#### Changes in chlorophyll pigment contents and gas exchange parameters

Diploid leaves produced significantly lower contents of total Chl, Chl a and Chl a/Chl b under 500 mM NaCl treatment compared with controls. A higher Chl b content was detected under 500 mM NaCl treatment; however, this difference was not statistically significant. No significant changes were found in total Chl, Chl a and Chl b contents under 250 mM NaCl (Table 2). 4× leaves contained less total Chl, Chl a and Chl a/Chl b under 250 mM NaCl treatment; higher contents of total Chl, Chl a and Chl b and lower contents of Chl a/Chl b were observed under 500 mM NaCl treatment (Table 2). In 2× leaves, there were significant decreases in the photosynthetic rate (P_n_), stomatal conductance (G_s_) and the leaf intercellular CO_2_ concentration (C_i_) under 250 mM NaCl treatment. These measurements decreased further under 500 mM NaCl treatment (Table 2). However, NaCl treatment did not significantly affect P_n_, G_s_ or C_i_ in 4× leaves (Table 2).

#### Analysis of protein expression changes under salt treatment

**Figure 4:**
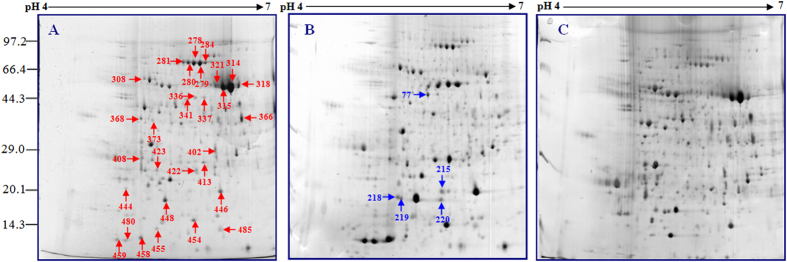
Coomassie Brilliant Blue (CBB)-stained two-dimensional electrophoresis gels of proteins from chloroplasts of 2× under NaCl stress using 2D Gels Analysis. Proteins were separated on 13 cm IPG strip (pH 4-7 linear gradient) by isoelectric focusing, followed by 12.5% SDS-PAGE (sodium dodecyl sulfate polyacrylamide gel electrophoresis).

**Figure 5:**
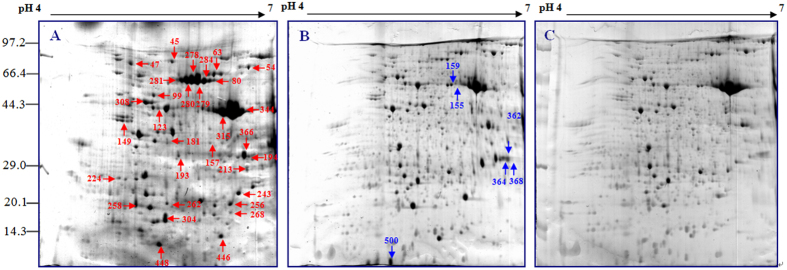
Coomassie Brilliant Blue (CBB)-stained two-dimensional electrophoresis gels of proteins from chloroplasts of 4× under NaCl stress using 2D Gels Analysis. Proteins were separated on 13 cm IPG strip (pH 4-7 linear gradient) by isoelectric focusing, followed by 12.5% SDS-PAGE (sodium dodecyl sulfate polyacrylamide gel electrophoresis).

To investigate changes in protein profiles in 2× and 4× leaves after salt treatment, a comparative proteomic analysis was performed. The representative gel images of 2× and 4× leaves are shown, and the positions of the identified expressed spots are marked in Figs 4 and 5. For each gel, more than 800 spots were reproducibly detected. These proteins were well separated in 2D gels. The isoelectric points (pI) were from 4.7 to 6.8, and molecular masses were from 14 to 90 kDa.

**Figure 6:**
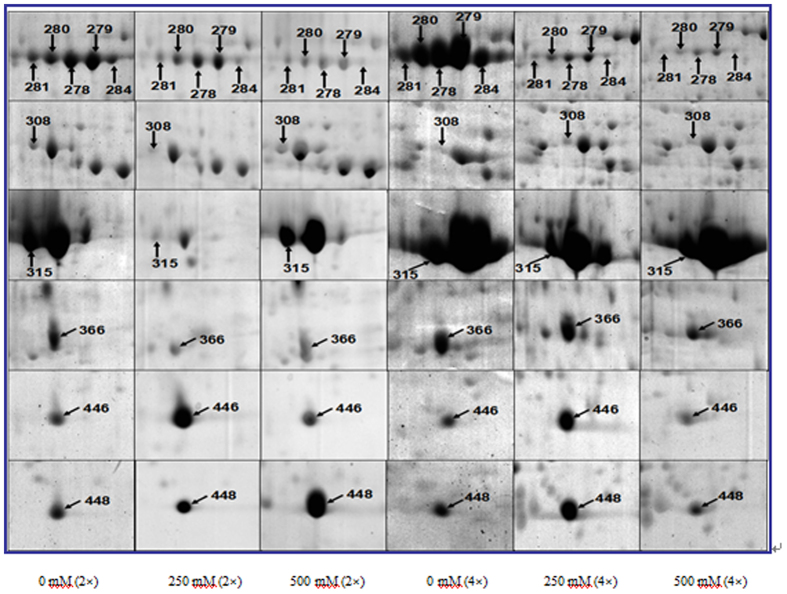
Common protein spots of 2× (**A**) and 4× (**B**) black locust leaves after 7 days of treatment under 0, 250, and 500 mM NaCl, respectively.

A total of 62 spots in 2× and 4× leaves exhibited reproducible and significant (>2.5-fold and p < 0.05) changes under salt stress. Of these, 25 protein spots were differentially expressed only in 2× leaves under salt stress. In the leaves of 2× plants treated with 250 mM NaCl, 10 proteins were up-regulated, 8 were down-regulated, 1 disappeared and 6 were specifically induced. In the leaves of 2× plants treated with 500 mM NaCl, 0 proteins were up-regulated, 11 were down-regulated, 4 did not exhibit significant change, 8 disappeared and 2 were detected specifically (Table 3). In the leaves of 4× plants treated with 250 mM NaCl, there were 27 spots differentially expressed under salt stress. Of these, 7 proteins were up-regulated, 1 did not exhibit significant change, 14 were down-regulated and 5 were produced specifically. In the leaves of 4× plants treated with 500 mM NaCl, 7 proteins were up-regulated, 13 were down-regulated, 2 did not show significant changes, 1 disappeared and 4 were induced. In addition, 10 proteins overlapped in both 2× and 4× plants under salt stress; these are enlarged in Fig. 6.

#### Identification of Differentially Expressed Proteins under Salt Treatment

To further identify the proteins differentially expressed under salt treatment, these proteins were excised, digested and analyzed based on information from BLAST alignments, Gene Ontology analysis and the literature. The identified proteins cover a wide range of molecular functions and were grouped into the following 7 functional categories: photosynthetic Calvin-Benson Cycle (26), photosynthetic electron transfer (7), regulation/defense (5), chaperone (3), energy and metabolism (12), redox homeostasis (1) and unknown function (8) (Table 4 and Table S4).

#### Western Blot Analysis

**Figure 7:**
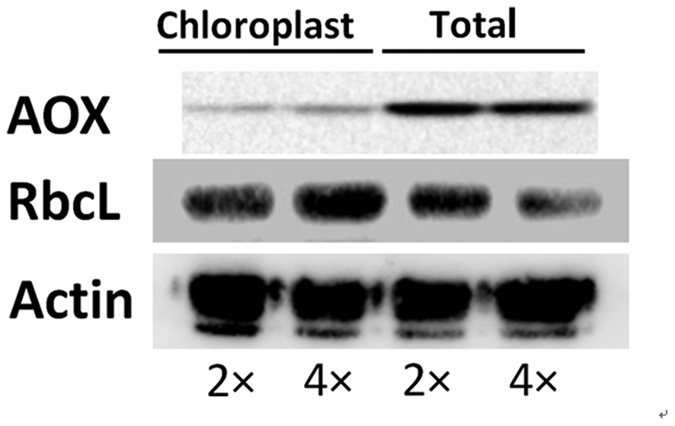
Western blot of alternative oxidase (AOX), ribulose-1,5-bisphosphate carboxylase/oxygenase (RbcL) and *β*-actin in chloroplast protein fraction (Chloroplast) and total protein (Total) extracts from controls of 2× and 4× leaves.

To estimate protein contaminant in our chloroplast isolation, a specific antibody against mitochondrial AOX was used to assess mitochondria contamination because this organelle is the common contaminant of chloroplast protein preparations. As the result shown in Fig. 7, there was a very weak band detected in the chloroplast protein fractions, compared with the signals in the total protein extracts, indicating that only small mitochondrial contaminants were present in our chloroplast preparations. Additionally, the signals from antibodies against RcbL recognized proteins from the both chloroplast protein fractions and the total protein extracts, were strong in the chloroplast fractions (Fig. 7). These results suggested that the chloroplast protein extracts were relatively highly purified in our study.

#### Quantitative Real Time PCR

**Figure 8:**
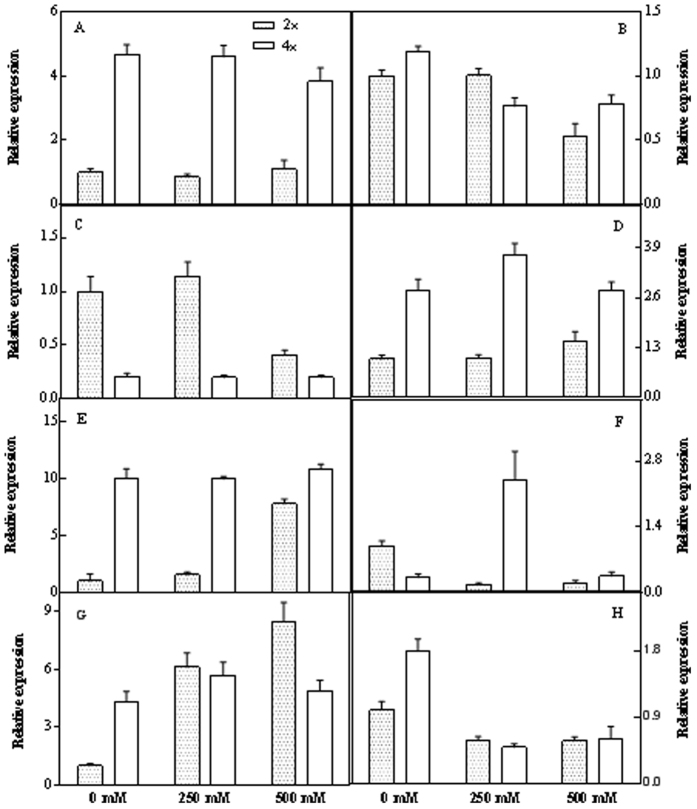
Expression of eight salt stress related genes including *ASCF1* (**A**), *FBA2*(**B**), *LADP* (**C**), *TRKT* (**D**), *HSP* (**E**), *LETM* (**F**), *FTM* (**G**) and *AAT* (**H**) of 2× and 4× black locust leaves after 7 days of treatment under 0, 250, and 500 mM NaCl, respectively. The genes are listed in Supplemental Table S1 and Table S2.

To investigate the relationship between the transcriptional and translational levels of responsive genes to salt stress, we employed qRT-PCR to analyze 20 genes based on the proteomics data. In the result, 19 genes of the 20 selected genes were polymerised successfully, only one gene, Lr10 (spot. 280) was failure (Supplemental Fig. 1). Among the nineteen genes, qRT-PCR analysis revealed that eight of them showed similar transcriptional expression trends with their protein expression pattern observed in 2-DE results, i.e. *ASCF1* (spot. 308), *FBA2* (spot. 366), *LADP* (spot. 280), *TRKT* (spot. 63), *HSP* (spot. 47), *LETN* (spot. 422), *FTN* (spot. 423), *AAT* (spot. 155) (Fig. 8). However, the other eleven genes showed different transcriptional expression trends compared with their protein expression pattern observed in 2-DE results (Supplemental Fig. S2).

## Discussion

It is regret that we have no any direct evidence that increase in nuclear genome ploidy could affect chloroplast function. However, some studied showed that there is relationship between the ploidy level of plants and the number of chloroplast in stomatal guard cells in *Acacia mearnsii*^14^ and *Brassica oleracea*^15^. In addition, our previous study showed that salt stress resulted in distorted chloroplasts, swollen thylakoid membranes, accumulation of plastoglobules, and increased starch grains in diploid black locust compared to those in tetraploid black locust^16^. Therefore, there is direct or indirect relationship between nuclear genome ploidy and chloroplast function.

How plants respond to salt stress depends on genotype, the severity of salinity and the stage of development^17^. Here, the responses of diploid and tetraploid black locust (*Robinia pseudoacacia* L.) plants under moderate (250 mM NaCl) and severe (500 mM NaCl) salt stress were investigated. Generally, isolated organelles under salt stress accumulate increased amounts of ROS, such as H_2_O_2_. Like the stable superoxide anion, H_2_O_2_ may act as a signal molecule during stress responses and can induce the expression of genes encoding antioxidant enzymes related to stress tolerance^18^,^19^,^20^,^21^. In this study, the higher H_2_O_2_ level observed in chloroplasts of 4× leaves compared with those of 2× leaves (Table 1) indicated that H_2_O_2_ accumulation may induce increases in APX, DHAR and MDHAR activities in 4× leaves (Fig. 2). SOD, the first line of defense against oxidative stress, had higher activity levels in salt-stressed 4× leaves compared to 2× leaves (Fig. 2). Four key enzymatic and non-enzymatic antioxidants in the Halliwell-Asada pathway, AsA, GSH, GR, and GST, exhibited higher activities in 4× leaves compared to 2× after salt treatment (Fig. 2 and Table 1). Taken together, these results indicate that the increased accumulation of H_2_O_2_ and increased levels of important antioxidative enzymes and non-enzymatic antioxidants in 4× plants compared to 2× plants under salt stress may alleviate oxidative stress in 4× plants. Furthermore, our proteomics results (see below) reveal that the expression levels of some regulation/defence proteins (spots 80 and 279) were decreased in 4× leaves under salt stress (Table 4). These findings suggest that 4× plants mainly relied on the Halliwell-Asada pathway to cope with salt stress.

Our study shows that salt stress had greater negative effects on photosynthesis in 2× plants compared to 4× plants under salt stress (Table 2). Photosynthetic capacity is determined by several factors, such as stomata, the availability of ATP and the activity of photosynthetic enzymes. Information concerning the net photosynthetic rate (P_n_), leaf intercellular CO_2_ concentration (C_i_) and stomatal conductance (G_s_) is important for understanding leaf physiological processes in nature. P_n_ is limited by both stomatal and non-stomatal factors. The stomatal factor is associated with decreased C_i_, which is caused by decreases in stomatal conductance G_s_^22^. Our study showed that salt stress decreased P_n_, G_s_ and C_i_ in 2× and 4× plants (Table 2). However, these reductions were less severe in 4× plants compared to 2× plants. These results indicate that 4× plants were better able to acclimate to salt stress. Similar results have been reported in our previous studies^16^. A decrease in G_s_ is often a factor in decreases in P_n_ due to the concomitant decrease in C_i_.

Chlorophyll content acts as an index of leaf senescence, as chlorophyll is fundamental to photosynthesis. Different chlorophyll contents from different polyploid plants under environmental stress have been previously observed^23^. Previous studies indicated that salinity decreased chlorophyll pigment contents^24^. In accordance with these previous reports, lower contents of total Chl and Chl a as well as a lower Chl a/Chl b ratio were observed in salt-stressed 2× plants compared to the controls. Increases in total Chl, Chl a and Chl b coincided with a much milder decline in P_n_ in 4× plants under 500 mM NaCl treatment. These results showed that 2× plants suffer from greater negative effects than 4× plants when exposed to salt stress.

Salinity can affect cell ultrastructure in many plant species. In plant cells, chloroplasts are an important site of photosynthesis and other biosynthetic pathways^25^. Salinity can change the integrity and functionality of chloroplasts, which in turn affect cell functionality^26^. Our results confirm these observations and reveal that chloroplasts in salt-stressed 2× plants were incompact and swollen in structure; a decrease in the number of grana (Gr) was also observed. Salt stress induced less serious changes in the ultrastructure of chloroplasts in 4× plants. These structural changes lower the photosynthetic efficiency of plants, further leading to structural disruptions. According to our results, salt stress led to a decrease in P_n_ in 2× plants but had lesser effects on 4× plants. These findings suggest that 4× plants possess a better self-protection mechanism in chloroplasts under salt stress than 2× plants.

As expected, the largest functional group of proteins identified was related to the photosynthetic Calvin-Benson Cycle. This cycle is known as the photosynthetic carbon reduction pathway and provides substrates for the synthesis of sucrose, starch and proteins^27^. Enzymes of the Calvin-Benson Cycle are located in the chloroplast stroma. Diverse changes in these enzymes have been observed among different plant species after salt treatment. In this study, 26 proteins were identified as belong to the Calvin-Benson cycle (Table 4).

Ribulose bisphosphate carboxylase/oxygenase (Rubisco) is an enzyme complex in plants that is comprised of 8 large catalytic subunits (LSU) and 8 small subunits (SSU). In this study, the experimental molecular masses of some of proteins (e.g., spots 99, 215, 218, 219, 220, 243 and so on) were lower than their theoretical values. This could be due to protein degradation or may result from a larger pool of Rubisco being made or turned over. In 2× plants, the Rubisco large subunit (e.g., spots 215, 220 and 455) and the Ribulose bisphosphate carboxylase large chain (e.g., spots 218, 219, 454, 458, 459 and 480) were induced only under 250 mM NaCl treatment; these spots were reduced or eliminated under 500 mM NaCl treatment, suggesting that 2× plants can better tolerate milder salt stress. By contrast, the abundance of the Rubisco large subunit (spots 304 and 344) increased or changed little (spots 446 and 448) in 4× plants compared to 2× plants under NaCl treatment. Increases in microvariations of these proteins may contribute to salt tolerance in 4× plants.

The content of Rubisco activase decreased in 4× plants (Table 4). Rubisco activase is an ATPase protein that makes the active site of Rubisco catalytically competent by carbamylation with CO_2_. Overexpression of Rubisco activase decreases photosynthetic CO_2_ assimilation capacity by reducing the Rubisco content in rice leaves^28^. *Aeluropus lagopoides* (Poaceae), a halophyte C_4_ plant, also showed severe down-regulation of Rubisco activase under salinity stress^29^. In this study, the down-regulation of Rubisco activase levels in 4× plants under salt stress may alleviate a decrease in Calvin-Benson cycle activity. This would sustain reasonable photosynthetic CO_2_ assimilation capacity by lightening energy expenditures to strengthen salt tolerance.

It is well known that the photosynthetic control of electron transport is fundamental to the regulation of photosynthesis^18^. When plants are exposed to salt stress, electron transport may be inhibited and photoinhibition occurs as a consequence. ATP synthase produces ATP from ADP via a proton gradient across a membrane, and ATP synthase subunits (alpha, beta and delta) are involved in the photosystem electron transfer reaction^30^. Chlorophyll a/b-binding (CAB) proteins, important components of the major light-harvesting complex, are the apoproteins of photosystem II (PSII)^31^.

In 4× plants, ATP synthase CF1 beta subunit (spot 123), ATP synthase beta subunit (spot 278) and chlorophyll a/b-binding 3C-like protein (spot 258) decreased in abundance after salt treatment (Table 4). However, no significant decrease in P_n_ was observed in salt-stressed 4× plants (Table 2). These findings suggest that photosynthesis in 4× plants is not susceptible to salt stress. Alternatively, these three subunits do not play a role in protecting ATP synthase against salt stress and cannot provide energy for maintaining normal physiological processes in 4× plants. In other words, this result is intriguing and suggests that 4× plants may use different strategies to respond to salt stress. In 2× plants, three proteins (spots 278, 408 and 444) were also down-regulated after salt stress. However, spot 308 was up-regulated dramatically after 500 mM NaCl treatment. This indicates that the mechanisms that protect plants from salt stress are different in 2× and 4× plants.

Many disease or pathogenesis-related proteins (PR proteins) are involved in plant responses to environmental stressors. These proteins are believed to play important roles in the plant’s defense against abiotic stressors. Leaf rust resistance genes can provide the most durable resistance to leaf rust in wheat throughout the world. Their precise response mechanisms, specifically in salt tolerance, are unclear. In our study, leaf rust resistance protein (spots 80 and 279), L-ascorbate peroxidase (spot 413), lectin (spot 422) and ferritin-3 (spot 423) changed in abundance after NaCl treatment. Among these regulation/defense-related proteins, two proteins (spots 422 and 423) were increased in 2× plants treated with 250 mM NaCl. This suggests that such proteins may decrease the risks to the plant under moderate salinity conditions. Our results are consistent with previous results. For example, Sun, Yu^32^ found that lectin protein kinase is a positive regulator of tolerance to salt stress in *Arabidopsis*. In pear (*Pyrus pyrifolia* ‘cuiguan’), ferritin genes accumulated under abiotic stresses^33^. In contrast, L-ascorbate peroxidase (spot 413) decreased in abundance after salt treatment; however, ascorbate peroxidase (APX) accumulated slightly in salt-treated 2× plants (Fig. 2B). APXs detoxify peroxides such as hydrogen peroxide using ascorbate as a substrate. These enzymes play crucial roles in protecting against oxidative stress^34^. This suggests that 2× plants did not primarily rely on L-ascorbate peroxidase to address salt stress.

Chaperone proteins are involved in protein folding, translocation and degradation. These proteins also play an important role in protecting plants against environmental stressors^35^. In our study, chaperones were found to be significantly down-regulated under salt stress. These proteins include chaperone protein ClpC, chloroplastic-like isoform 1 (spot 45), heat shock protein (spot 47) and chlorophyllase-2, chloroplast precursor (spot 280). Decreases in these proteins may reduce the stability of photosynthetic complexes. Velikova, Ghirardo^26^ observed that some chloroplast proteins, such as chaperonins, were significantly down-regulated in poplar plants. By contrast, chaperone protein ClpC was induced by cold and salt stress^36^. Previous studies have shown that increased expression of heat shock proteins can protect plants against stress-induced damage^37^. Here, a heat shock protein (spot 47) was down-regulated in 2× and 4× plants under salt stress (Table 4). Therefore, the function of chaperone proteins in salt tolerance deserves further attention in future studies. In other words, our result suggested that 4× plants might tolerate salt stress via chaperone proteins.

Salt stress can result in energy and metabolism changes in plants. Such changes perturb osmotic and water homeostasis in plants^38^. In response, plants require energy and metabolism to regulate these processes. In this work, we found that the contents of more proteins involved in energy and metabolism were changed in 4× plants compared to 2× plants under salt tress (Table 4).

In 4× plants, proteins such as those represented by spots 63, 159, 181, 194 and 262 accumulated in response to salt treatment (Table 4). As previously reported, enolase (spot 159) is responsive to environmental stressors such as salt stress, drought, cold and anaerobic stress in many plant species^39^. Moreover, malate dehydrogenase (spot 194) and glutamine synthetase (spot 181) were also up-regulated by salt stress. In plants, malate dehydrogenase provides malate for C_4_ metabolism, pH balance, stomatal and pulvinar movement, respiration, the *β*-oxidation of fatty acids, and the functioning of legume root nodules^40^. Kalifa, Perlson^41^ also reported that the enzymatic activity of malate dehydrogenase increased when root nodules of tobacco were exposed to salt stress. The function of malate dehydrogenase suggests that 4× plants can cope with salt stress by accumulation of malate dehydrogenase. Glutamine synthetase is the first enzyme in the main pathway of ammonium assimilation in higher plants^42^. Hoshida, Tanaka^43^ found that over-expression of chloroplast glutamine synthetase in transgenic rice enhanced tolerance to salt stress. Increases in these proteins may provide sufficient energy and materials to allow 4× plants to resist salt stress.

Proteins involved in energy and metabolism, such as pyruvate short chain alcohol dehydrogenase (spot 281), tasselseed2-like short-chain dehydrogenase/reductase (spot 284), and pyruvate dehydrogenase (spots 373 and 402), showed reduction in levels in 2× plants after salt treatment. Pyruvate dehydrogenase, which is often considered a primary target for the adverse effect of many environmental stressors, was markedly down-regulated after salt treatment in 2× plants (Table 4). Disruption of the activity of this enzyme may lead to cellular damage and death^44^. Induction of this enzyme has also been reported in both the roots and shoots of mungbean (Vigna radiata L. Wilczek) seedlings coping with environmental stress^45^. Therefore, pyruvate dehydrogenase may be a positive regulator that mediates anti-stress processes in plants. Conversely, the inhibition of plant growth in 2× plants could be explained by a decrease in pyruvate dehydrogenase activity.

Additionally, salt stress led to decreases in the abundance of two proteins [short chain alcohol dehydrogenase, putative (spot 281) and tasselseed2-like short-chain dedydrogenase/reductase (spot 284)] in both 2× and 4× plants. Alcohol dehydrogenase, a well-known stress marker gene, can help detoxify highly reactive and toxic molecules that can otherwise attack proteins, nucleic acids and carbohydrates^46^. However, increases in alcohol dehydrogenase due to salt stress might inhibit plant growth and development^27^. Here, the decrease in alcohol dehydrogenase observed in 4× plants was greater than that in 2× plants under salt stress. Therefore, a decrease in this protein can promote plant growth and development in 4× plants under salt stress.

Here, proteins levels and gene transcription levels showed consistent or inconsistent (Fig. 7, Supplemental Fig. 1, Supplemental Fig. 2 and Table 4). This consistency between transcription levels of several of the proteins and protein expression changes suggested that these proteins may be firstly regulated at the transcriptional level after salt treatment. Additionally, this inconsistency between the protein and transcription levels could be due to post-translational processing or post-transcriptional regulation^47^,^48^. This inconsistency has been found by some previous studies^49^,^50^.

## Materials and Methods

### Plant materials and salt treatment

All materials were introduced directly to China from South Korea by Beijing Forestry University. Diploid and tetraploid black locust (*Robinia pseudoacacia* L.) plants have the same genetic origin^23^,^26^. Thirty (2-year-old) uniform plants were planted in plastic pots (21 cm in diameter and 21 cm in depth) filled with a 2:1 (v/v) mixture of soil and sand. The experiments were carried out at Harbin Experimental Forest Farm at Northeast Forestry University in June 2013. Potted plants were grown in the greenhouse (day/night air temperatures were 28/22 °C, the photoperiod was 12 h, and the relative humidity was 65–85%). Plant roots were treated with 250 mM (moderate salt stress) or 500 mM NaCl (high salt stress). To maintain a stable NaCl concentration, the solutions were changed daily. We will describe the salt treatment in more detail after 7 days so that it can be duplicated. After 7 days of treatment, fresh tissues from fully expanded leaves (the third to fifth leaves from the shoot apex) were collected for physiological measurements and transmission electron microscopy. Additional leaves were immediately frozen in liquid nitrogen and stored at −80 °C prior to chloroplast extraction. Three independent biological experiments were performed for each treatment.

### Physiological investigations

#### Enzymatic activity measurements

Superoxide dismutase (SOD. EC1.15.1.1) activity was measured using the method described by Beauchamp and Fridovich^51^. The reaction mixture contained 20 μL enzyme extract, 50 mM sodium phosphate buffer (pH 7.8), 100 μM ethylenediaminetetraacetic acid (EDTA), and 10 mM pyrogallol. Enzyme activity was detected spectrophotometrically at 420 nm. Glutathione reductase (GR, EC 1.6.4.2) activity was determined by nicotinamide adenine dinucleotide phosphate (NADPH) oxidation at 340 nm. The reaction mixture contained 10 μL enzyme extract, 100 mM potassium phosphate buffer (pH 7.8), 0.2 mM NADPH, 2 mM EDTA, and 0.5 mM glutathione. The reaction was initiated by adding NADPH at 25 °C. Ascorbate peroxidase (APX, EC1.11.1.11) activity was assayed using the method described by Nakano and Asada^52^. The reaction mixture contained 50 mM sodium phosphate buffer (pH 7) including 0.2 mM EDTA, 0.5 mM ascorbic acid, and 50 mg bovine serum albumin (BSA). The reaction was started by adding H_2_O_2_ at a final concentration of 0.1 mM. Glutathione-S-transferase (GST) was determined according to the method described by Neuburger, Journet^53^. Dehydroascorbate reductase (DHAR, EC 1.8.5.1) was measured following ascorbate (ASA) formation at 265 nm in a reaction solution containing 0.5 mM docosahexaenoic acid (DHA) and 5 mM reduced glutathione (GSH)^54^. Monodehydroascorbate reductase (MDHAR, EC 1. 6. 5. 4) was determined by measuring NADH (a reduced form of NADPH) oxidation at 340 nm. The reaction mixture contained 0.2 mM (NADH), 1 mM ASA, and 1 unit of ASA oxidase^55^.

#### Non-enzymatic antioxidant and H_2_O_2_ measurements

AsA (ascorbic acid) contents were determined according to Law, Charles^56^ with minor modifications. The reaction mixture included 0.2 ml extraction, 0.5 ml phosphate buffer (150 mM, pH 7.4), and 0.2 ml double-distilled water. Then, 0.4 ml α′-dipyridyl in 70% ethanol and 0.2 ml FeCl_3_ (3%) were added to the reaction solution. The mixtures were incubated at 40 °C for approximately 40 min. After centrifugation at 12,000 × g for 10 min, the clear supernatant was collected. The change in absorbance at 525 nm was monitored.

GSH determination was carried out according to the method described by Ellman^57^. The absorbance of reduced chromogen and 5,5′-Dithiobis (2-nitrobenzoic acid) (DTNB) was measured at 412 nm. This measurement was used to determine GSH concentration.

Hydrogen peroxide (H_2_O_2_) was detected spectrophotometrically according to Sergiev, Lavrik^58^. The supernatant was homogenized in 0.1% TCA in an ice bath. After centrifugation at 12,000 × g for 10 min, 0.5 ml extraction solution was mixed with 0.5 ml potassium phosphate buffer (pH 7.5) and 1 ml potassium iodide (1 M). The absorbance of the supernatant was measured at 390 nm. The concentration of H_2_O_2_ was obtained using a standard curve.

#### Transmission electron microscopy

Fresh leaves approximately 1.5 cm^2^ in size were sampled, fixed immediately with 2.5% (v/v) glutaral pentanedial at 4 °C for 2 h, and washed twice in 0.1 M PBS (sodium phosphate buffer, pH 6.8) at 4 °C. They were then post fixed in 2% osmium tetraoxide (O_s_O_4_) for 2 h. Samples were then sequentially dehydrated with 50, 70, 90, and 100% acetone and embedded in Epon 812 for 2 h. Ultrathin sections (70 nm) were sliced, stained with uranyl acetate and lead citrate, and then mounted on copper grids for viewing on a H-600 IV TEM (Hitachi, Tokyo, Japan) at an accelerating voltage of 60 kV.

#### Measurement of chlorophyll pigment and gas exchange parameters

For chlorophyll pigment determination, 0.3 g leaves were ground in 80% cold acetone and centrifuged at 12,000 × g for 20 min. Then, the supernatant was collected and diluted in 10 ml acetone, and the absorbance at 645 nm and 663 nm was monitored (Mehrnaz Hamami and Ghorbanpour 2013). Gas exchange parameters were measured from 09:00 to 11:30 in the morning. Net photosynthetic rate (P_n_), stomatal conductance (G_s_), and intercellular CO_2_ (C_i_) were measured with a Li-Cor 6400 portable photosynthesis measuring system (LI-Cor Inc, Lincoln, NE) at 7 days after treatment with 0, 250 mM or 500 mM NaCl. The measurement conditions were as follows: leaf temperature was 25 °C, the photon flux density (PFD) was 900 μmol m^−2^ s^−1^, the relative air humidity was 70%, and the ambient CO_2_ concentration was 450 μmol^−1^.

### Two-dimensional gel electrophoresis

#### Chloroplast isolation

To isolate and purify chloroplasts from leaves, we followed protocols described by with minor modifications. All steps were carried out at 4 °C. A 30 g sample of leaves was harvested and ground in 200 ml isolation buffer I, which contained 50 mM HEPES/KOH (pH 7.5), 5 mM hexanoic acid, 0.3% BSA (w/v), 0.3 M sucrose, 10 mM β-mercaptoethanol, 20 mM EDTA, 30 mM Na-ascorbate and 1% (w/v) PVP. The homogenate was then filtered through six layers of mesh nylon cloth (40 × 40 μm) and centrifuged at 4,000 × g for 10 min. The supernatant was centrifuged at 20,000 × g for 10 min. The precipitate was carefully suspended in buffer II, which contained 20 mM HEPES/KOH (pH 7.5), 330 mM sorbitol, 10 mM NaCl, 2 mM EDTA and 5 mM Na-ascorbate, and washed twice. Subsequently, the re-suspended chloroplasts were loaded onto a percoll gradient consisting of 6:6:6:3 ratios, top to bottom, of 10, 40, 70 and 90% percoll. The mixture was centrifuged for 0.5 h at 40,000 × g. Chloroplasts were present between the 40 and 70% interface. Then, the intact chloroplasts were collected, washed and centrifuged at 10, 000 × g for 15 min in buffer II.

Chloroplast proteins were extracted by adding 0.7 ml 10% acetone to the tube, which was then stored at −20 °C for 12 h. Then, samples were centrifuged at 25,000 × g for 15 min. The precipitate was washed with 80% then 100% cold acetone and centrifuged for 30 min. After centrifugation, the precipitate was vacuum dried. Then, the dried powder was dissolved in an IEF buffer containing 7 M urea, 2 M thiourea, 4% 3-[(3-cholamidopropyl)-dimethylammonio]-1-propane sulfonate (CHAPS), 40 μM dithiothreitol (DTT) and 0.2% pharmalytes (pH 4–7). The protein solution was stored at −80 °C until use. The protein concentration was determined using the method described by Bradford^59^.

#### Gel electrophoresis and gel staining

400 μg protein samples were rehydrated in 250 μl protein rehydration solution and used for isoelectric focusing (IEF). Subsequently, the IPG strips (13 cm, pH 4–7) were incubated for 12 h at room temperature. The IEF procedure consisted of the application of 30 V for 14 h, 100 V for 1 h, 500 V for 1 h, 1000 V for 1 h, 8000 V for 1 h and 8000 V for 5 h. After IEF, gels were equilibrated in 10 ml equilibration buffer containing 6 mM urea, 50 μM Tris-HCl (pH 8.8), 2% (w/v) SDS, 30% glycerine and 2% (w/v) DTT for 15 min. Gels were then incubated in a similar solution containing 1.5% iodoacetamide instead of DTT for 15 min. Second dimension SDS-PAGE was conducted using a 12.5% (w/v) polyacrylamide gel. After electrophoresis, the gels were stained with Coomassie brilliant blue (CBB) R-250 solution containing 25% methanol, 8% acetic acid and 0.1% (w/v) CBB until protein spots were clearly visible.

#### Gel Image Analysis and Matrix-Assisted Time of Flight Mass Spectroscopy (MALDI-TOF-MS) Analysis

Gel images were scanned using an ImageScanner III (GE Healthcare, Bio-Sciences, Uppsala, Sweden). Images were analyzed using ImageMaster 2D Platinum 7.0 software (Amersham Biosciences, Piscataway, NJ, USA, 2011). The average volume percent values were calculated from three technical replicates to represent the final volume percent values of each biological replicate. The experimental molecular masses and isoelectric points (pI) of the protein spots were determined using 2-DE standards and the interpolation of missing values on the IPG strips. Spots were quantified based on the total density of the gels and the percentage volume. Significantly different spots, which were determined as p < 0.05 and a change of more than 2.5-fold in abundance, were considered to be differentially accumulated proteins. Such proteins had to be consistently present in three replications.

Selected protein spots were excised, washed with 50% (v/v) acetonitrile in 0.1 M NH_4_HCO_3_, and dried at room temperature. Proteins were reduced with 1 mM DTT and 2 mM NH_4_HCO_3_ at 55 °C for 1 h and then alkylated with 55 mM iodoacetamide in 25 mM NH_4_HCO_3_ in the dark at room temperature for 45 min. The gel pieces were thoroughly washed with 25 mM NH_4_HCO_3_, 50% acetonitrile, and 100% acetonitrile, and then dried. The proteins were digested in 10 ml modified trypsin (Promega, Madison, WI, USA) solution (1 ng/ml in 25 mM NH_4_HCO_3_) during an overnight incubation at 37 °C. Digests were immediately spotted onto 600 mm anchorchips (Bruker Daltonics, Bremen, Germany). Spotting was achieved by pipetting 1 ml analyte onto the MALDI target plate in duplicate and then adding 0.05 ml 20 mg/ml α-CHCA in 0.1% TFA/33% (v/v) ACN, which contained 2 mM ammonium phosphate. All samples were analyzed in the positive-ion reflection mode on a TOF Ultraflex II mass spectrometer (Bruker Daltonics, Billerica, United states). Each acquired mass spectra (a m/z range of 700–4000 and a resolution of 15,000–20,000) was processed using FlexAnalysis v2.4 software (Bruker Daltonics, Bremen, Germeny, 2004). Proteins were identified with Mascot software (http://www.matrixscience.com) based on the mass signals used to search for proteins in the SwissProt, NCBInr, and MSDB databases.

#### Western Blot Analysis

To estimate protein contaminants in the chloroplast isolated, western blot analysis was performed according to Wang, Liang^60^ with minor modification. In brief, equal amount of chloroplast protein or total protein (prepared as previous, Wang, Liang^60^) of black locust leaves (2× and 4×) were resolved on 12% SDS-PAGE and transferred onto nitrocellulose membrane (GE Healthcare, UK). The membranes were blocked with TBST buffer (10 mM Tris-HCl, pH 7.5, 150 mM NaCl, 0.05% Tween 20) containing 5% milk for 2 hours and then incubated with specific antibodies (Agrisera, Sweden) in TBST for 1 hour, including alternative oxidase (AOX, as a mitochondrial marker, ribulose-1,5-bisphosphate carboxylase/oxygenase (RuBisCO) large subunit (RbcL, as a chloroplast marker) and β-actin. After washing 3 times, the membrane was incubated with goat-anti-rabbit IgG secondary antibody conjugated to HRP (KPL, USA) diluted 1:10,000 in TBST for 1 h. Proteins were detected with enhanced chemiluminescence (ECL) reagents (Agrisera, Sweden) by ImageQuant Las 500 (GE, USA). Western blot analysis experiments were repeated at least three times, and the representative data are shown.

#### Quantitative Real Time PCR

To investigate the relationship between the transcriptional and translational levels of salt stress related genes, we employed qRT-PCR for 20 genes selected based on the proteomics results (Supplemental Table S1). Total RNA was isolated using a plant RNA extraction kit (Bioteke, China) and cDNA was synthesized from 1 μg of the total RNA with PrimeScript Reverse Transcriptase (Takara, Japan) according to the manufacturer’s instructions. Specific primer pairs for the selected genes were designed by comparing the nucleotide sequence of conserved region using BioEdit, Premier 5.0 and Oligo 6.0 (Supplemental Table S2). The qRT-PCR was performed using the SYBR Green Realtime PCR Master Mix (Toyobo, Japan) with Lightlycler480 (Roche, USA), based on semi-quantitative PCR to test the primer pairs and conform the annealing temperatures (Supplemental Table S2). The expression levels of the actin were used as an internal control (reference gene). Relative expression of the target genes was calculated using the comparative Ct method.

#### Statistical Analyses

Statistical analyses were performed with SPSS 17.0 software (SPSS Inc. Chicago, IL, USA, 2009). All parameters are presented as mean ± standard error and were obtained from at least three replicates. Parameters were analyzed using Duncan’s multiple range test or Student’s t-test. A p-value < 0.05 was considered significant.

## Additional Information

**How to cite this article**: Meng, F. *et al.* Physiological and proteomic responses to salt stress in chloroplasts of diploid and tetraploid black locust (*Robinia pseudoacacia* L.). *Sci. Rep.* **6**, 23098; doi: 10.1038/srep23098 (2016).

## Supplementary Material

Supplementary Information

## Figures and Tables

**Table 1:** Changes in the contents of AsA, GSH and H_2_O_2_ in chloroplasts of 2× and 4× leaves after salt treatment.

Treatment	AsA (μmol g^−1^ FW)	GSH (μmol g^−1^ FW)	H_2_O_2_ (nmol g^−1^ FW)
2×			
Control	0.37 ± 0.03b	1.25 ± 0.08c	11.0 ± 0.7b
250 mM	0.40 ± 0.08ba	1.73 ± 0.21b	11.0 ± 1.3b
500 mM	0.47 ± 0.02a	2.18 ± 0.20a	13.4 ± 0.4a
4×			
Control	0.38 ± 0.05c	1.88 ± 0.06b	15.9 ± 3.5b
250 mM	0.56 ± 0.02b	2.33 ± 0.11a	15.6 ± 2.2b
500 mM	0.90 ± 0.01a	2.67 ± 0.20a	21.8 ± 3.2a

**Table 2:** Changes in chlorophyll pigment contents and gas exchange parameters in chloroplast of 2× and 4× leaves after salt treatment.

Treatment	Chlorophyll (mg g^−1^FW)	Chlorophyll *a*(mg g^−1^FW)	Chlorophyll *b*(mg g^−1^FW)	chlorophyll *a*/*b*	*P*_*n*_(μmol m^−2^ s^−1^)	*G*_*s*_(mmol m^−2^ s^−1^)	*C*_*i*_(μmol CO_2_ mol^−1^)
2×							
Control	0.861 ± 0.015a	0.675 ± 0.023a	0.184 ± 0.015a	3.67 ± 0.03a	16.1 ± 2.31a	0.524 ± 0.043a	432 ± 62a
250 mM	0.761 ± 0.050a	0.526 ± 0.039a	0.233 ± 0.048a	2.26 ± 0.03b	12.2 ± 3.26b	0.313 ± 0.024b	333 ± 52b
500 mM	0.666 ± 0.012b	0.450 ± 0.013b	0.216 ± 0.012a	2.14 ± 0.01b	8.78 ± 2.24c	0.172 ± 0.056c	246 ± 28c
4×							
Control	0.926 ± 0.014a	0.631 ± 0.014a	0.216 ± 0.014b	2.92 ± 0.03a	17.9 ± 2.4a	0.599 ± 0.023a	513 ± 75a
250 mM	0.793 ± 0.090b	0.555 ± 0.063a	0.238 ± 0.067b	2.33 ± 0.02b	17.2 ± 1.3a	0.545 ± 0.135a	501 ± 26a
500 mM	1.032 ± 0.005a	0.699 ± 0.003a	0.333 ± 0.048a	2.10 ± 0.03b	15.2 ± 1.4a	0.410 ± 0.089a	487 ± 56a

**Table 3:** Number of differently expressed proteins pots under different salt treatment conditions.

	Up-regulated spots	Down-regulated spots	no significantly changed spots	not detected spots	newly detected spots
2×					
250 mM Vs 0 mM	10	8	0	1	6
500 mM Vs 0 mM	0	11	4	8	2
500 mM Vs 250 mM	3	11	0	12	1
4×					
250 mM Vs 0 mM	7	14	1	0	5
500 mM Vs 0 mM	7	13	2	1	4

**Table 4:**
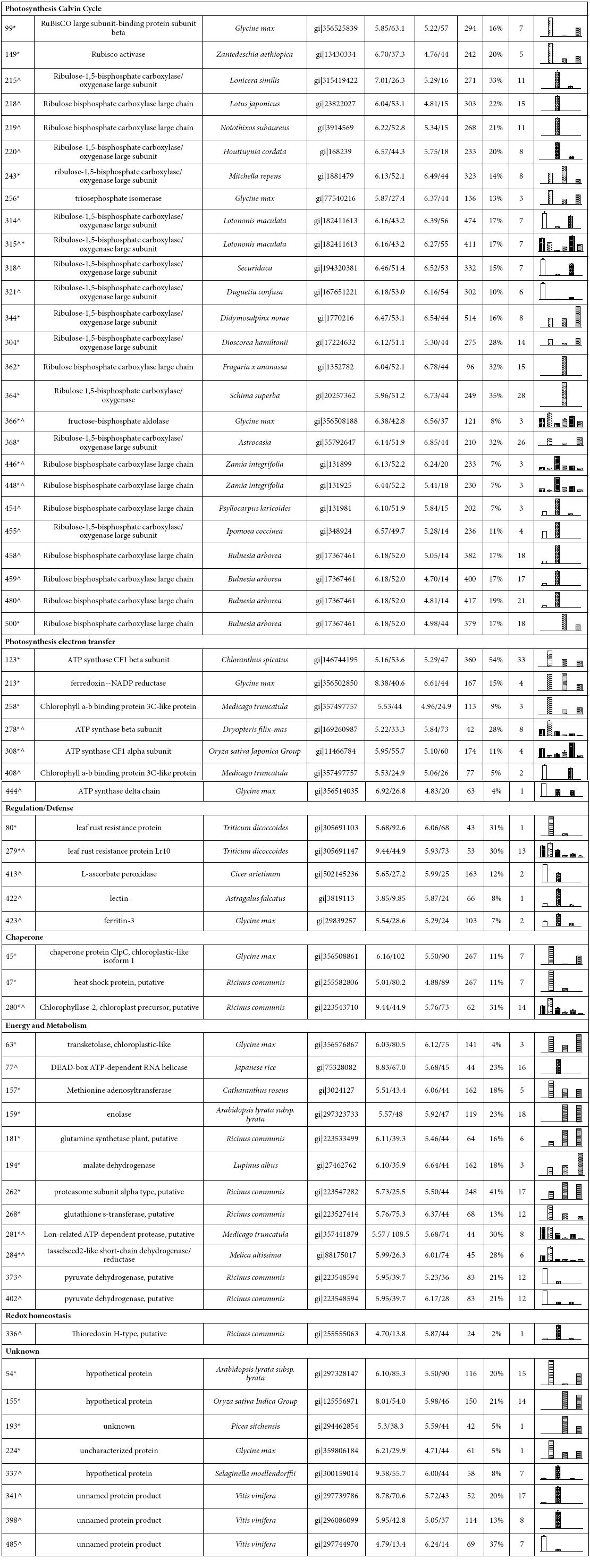
Differentially expressed proteins spots in chloroplasts between 2× and 4× under NaCl stress using 2D.

^a^Spot number as denoted in Figure. ^b^Accession number from NCBInr database. ^C^Theoretical molecular weight (KDa) and isoelectric point (pI). ^d^Experimental molecular weight (KDa) and isoelectric point (pI). ^e^Protein data were analyzed by searching against the NCBInr database and Matrix Science. ^f^percentage of predicated protein sequence covered by matched sequences. ^h^Mean of protein abundance and standard error. Six treatments including 2× and 4× (0, 250 mM and 500 mM NaCl for 7 days) were performed. ^proteins from 2× *proteins from 4×. *^proteins from 2× and 4×.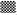, nonsalt stressed (0 mM NaCl) 2×.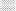, nonsalt stressed (0 mM NaCl) 4×.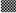, salt-stressed (250 mM NaCl) 2×.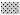, salt-stressed (250 mM NaCl) 4×.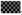, salt-stressed (500 mM NaCl) 2×.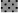, salt-stressed (500 mM NaCl) 4×.
