# Supplementary material for: Physiological and proteomic responses to salt stress in chloroplasts of diploid and tetraploid black locust (Robinia pseudoacacia L.)
Source: Sci Rep. 2016 Mar 15;6:23098. doi: 10.1038/srep23098 (PMC4791547; doi:10.1038/srep23098)
Supplement: Supplementary Information [file srep23098-s1.docx]

**Physiological and proteomic responses to salt stress in chloroplasts of diploid and tetraploid black locust (*Robinia pseudoacacia* L.)**

**Fanjuan Meng^1,#^, Qiuxiang Luo^2,#^, Qiuyu Wang^1^, Xiuli Zhang^1^, Zhenhua Qi^1^, Fuling Xu^1^,** **Xue Lei^1^, Yuan Cao^1^, Wah Soon Chow^3^, Guangyu Sun^1,﹡^**

**^1 College of Life Science, Northeast Forestry University, Harbin 150040, P. R. China,^**

**^2 Key Laboratory of Saline-Alkaline Vegetation Ecology Restoration in Oil Field (SAVER), Ministry of Education, Alkali Soil Natural Environmental Science Center, Northeast Forestry University, Harbin, 150040, P. R. China,^**

**^3 Division of Plant Science, Research School of Biology, The Australian National University, 46 Biology Place, Acton 2601, ACT, Australia^**

^# The first two authors contributed equally to this paper^

E-mail: mfj19751@163.com; fanjuanmeng2014@126.com


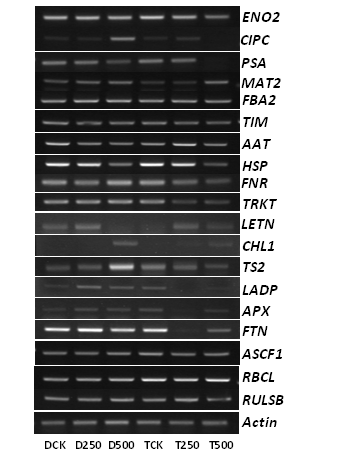


Figure S1. Semi-quantitative PCR analysis of nineteen genes including *ENO2*, *CIPC*, *PSA*, *MAT2*, *FBA2*, *TIM*, *AAT*, *HSP*, *FNR*, *TRKT*, *LETN*, *CHL1*, *TS2*, *LADP*, *APX*, *FTN*, *ASCF1*, *RBCL*, *RULSB* and *Actin* of 2× and 4× black locust leaves after 7 days of treatment under 0, 250, and 500 mM NaCl, respectively. DCK, 2× under 0 mM NaCl; D250, 2× under 250 mM NaCl; D500, 2× under 500 mM NaCl; TCK, 4× under 0 mM NaCl; T250, 4× under 250 mM NaCl; T500, 4× under 500 mM NaCl.

Figure S2. Expression levels of eleven salt stress related genes including *PSA* (A), *RULSB* (B), *FNR* (C), *RBCL* (D), *CHL2* (E), *TS2* (F), *ENO2* (G), *CIPC* (H), *MAT2* (I), *TIM* (J) and *APX* (K) of 2× and 4× black locust leaves after 7 days of treatment under 0, 250, and 500 mM NaCl, respectively. The genes are listed in Supplemental Table S1 and Table S2**.**

**Supplemental Table** **1 Differentially expressed proteins spots in chloroplasts between 2× and 4× under NaCl stress using 2D-Gel Analysis**

a Spot number as denoted in Fig 4 and Fig 5. bAccession number from NCBInr database. cTheoretical molecular weight (KDa) and isoelectric point (pI). dExperimental molecular weight (KDa) and isoelectric point (pI). eProtein data were analyzed by searching against the NCBInr database and Matrix Science. fpercentage of predicated protein sequence covered by matched sequences. hMean of protein abundance and standard error. Six treatments including 2× and 4× (0, 250 mM and 500 mM NaCl for 7 days) were performed. ^, proteins from 2× *, proteins from 4×. *^, proteins from 2× and 4×. , nonsalt stressed (0 mM NaCl) 2×. , nonsalt stressed (0 mM NaCl) 4×. , salt-stressed (250 mM NaCl) 2×. , salt-stressed (250 mM NaCl) 4×. , salt-stressed (500 mM NaCl) 2×. , salt-stressed (500 mM NaCl) 4×.

| **Spot No.^a^** | **Protein Name** | **Plant species** | **gi Number^b^** | **Thero pI/kDa^c^** | **Exper pI/kDa^d^** | | **Score^e^** | **SC^f^ (%)** | | **PN^g^** | **Peptide sequence** | | | | | | | **Observed m/z** | | **Expression pattern^h^** |
| --- | --- | --- | --- | --- | --- | --- | --- | --- | --- | --- | --- | --- | --- | --- | --- | --- | --- | --- | --- | --- |
| **Photosynthesis Calvin Cycle** | | | | | | | | | | | | | | | | | | | | |
| 99 | RuBisCO large subunit-binding protein subunit beta | *Glycine max* | [gi\|356525839](http://www.matrixscience.com/cgi/protein_view.pl?file=..%2Fdata%2F20131126%2FFTnorrcet.dat&hit=gi%7C356525839&db_idx=1&px=1&ave_thresh=46&_ignoreionsscorebelow=0&report=0&_sigthreshold=0.05&_msresflags=1025&_msresflags2=2&percolate=-1&percolate_rt=0&_minpeplen=7&sessionID=guest_guestsession) | 5.85/63.1 | 5.22/57 | | 294 | 16% | | 7 | K.LADLVGVTLGPK.G | | | | | | | 1182.7241 | |  |
|  |  |  |  |  |  | |  |  | |  | R.DLINILEDAIR.S | | | | | | | 1284.7367 | |  |
|  |  |  |  |  |  | |  |  | |  | R.GYISPYFVTDSEK.M | | | | | | | 1505.7314 | |  |
|  |  |  |  |  |  | |  |  | |  | K.AAVEEGIVVGGGCTLLR.L | | | | | | | 1700.9241 | |  |
|  |  |  |  |  |  | |  |  | |  | K.SQYLDDIAILTGGTVIR.E | | | | | | | 1835.0144 | |  |
|  |  |  |  |  |  | |  |  | |  | R.KSQYLDDIAILTGGTVIR.E | | | | | | | 1963.1091 | |  |
|  |  |  |  |  |  | |  |  | |  | K.TNDLAGDGTTTSVVLAQGLIAEGVK.V | | | | | | | 2430.2747 | |  |
| 149 | Rubisco activaseribulose bisphosphate carboxylase/oxygenase activase 1, chloroplastic-like | *Glycine max* | gi\|358248528 | 5.94/52.5 | 4.76/44 | | 235 | 12% | | 4 | K.FYWAPTR.D | | | | | | | 940.5079 | |    \|  \| \| --- \| |
| 215 | Ribulose-1,5-bisphosphate carboxylase/oxygenase large subunit | Lonicera similis | [gi\|315419422](http://gps/mascot/cgi/protein_view.pl?file=../data/20131225/F038258.dat&hit=1) | 7.01/26.3 | 5.29/16 | | 271 | 33% | | 11 | K.AQVETGEIK.G | | | | | | | 974.4501 | |  |
|  |  |  |  |  |  | |  |  | |  | K.DTDILAAFR.V | | | | | | | 1021.4697 | |  |
|  |  |  |  |  |  | |  |  | |  | K.DTDILAAFR.V | | | | | | | 1021.4697 | |  |
|  |  |  |  |  |  | |  |  | |  | K.LTYYTPDYETK.D | | | | | | | 1393.5828 | |  |
|  |  |  |  |  |  | |  |  | |  | K.LTYYTPDYETK.D | | | | | | | 1393.5828 | |  |
|  |  |  |  |  |  | |  |  | |  | R.LEDLRIPVAYVK.T | | | | | | | 1415.5615 | |  |
|  |  |  |  |  |  | |  |  | |  | K.TFQGPPHGIQVER.D | | | | | | | 1465.6761 | |  |
|  |  |  |  |  |  | |  |  | |  | K.TFQGPPHGIQVER.D | | | | | | | 1465.6761 | |  |
|  |  |  |  |  |  | |  |  | |  | K.YGRPLLGCTIKPK.L | | | | | | | 1502.7675 | |  |
|  |  |  |  |  |  | |  |  | |  | K.YGRPLLGCTIKPK.L | | | | | | | 1502.7675 | |  |
|  |  |  |  |  |  | |  |  | |  | R.DRFLFCAEALFK.A | | | | | | | 1516.7737 | |  |
| 218 | Ribulose bisphosphate carboxylase large chain | Lotus japonicus | [gi\|23822027](http://gps/mascot/cgi/protein_view.pl?file=../data/20131225/F038259.dat&hit=1) | 6.04/53.1 | 4.81/15 | | 303 | 22% | | 15 | K.AQEETGEIK.G | | | | | | | 1004.5218 | |    \|  \| \| --- \| |
|  |  |  |  |  |  | |  |  | |  | K.DTDILAAFR.V | | | | | | | 1021.4778 | |  |
|  |  |  |  |  |  | |  |  | |  | K.DTDILAAFR.V | | | | | | | 1021.4778 | |  |
|  |  |  |  |  |  | |  |  | |  | R.EITLGFVDLLR.D | | | | | | | 1275.6702 | |  |
|  |  |  |  |  |  | |  |  | |  | R.EITLGFVDLLR.D | | | | | | | 1275.6702 | |  |
|  |  |  |  |  |  | |  |  | |  | K.LTYYTPDYETK.D | | | | | | | 1393.6250 | |  |
|  |  |  |  |  |  | |  |  | |  | K.LTYYTPDYETK.D | | | | | | | 1393.6250 | |  |
|  |  |  |  |  |  | |  |  | |  | K.TFQGPPHGIQVER.D | | | | | | | 1465.6888 | |  |
|  |  |  |  |  |  | |  |  | |  | R.MSGGDHIHAGTVVGK.L | | | | | | | 1465.6888 | |  |
|  |  |  |  |  |  | |  |  | |  | K.EIKFEFQAMDTL.- + Oxidation (M) | | | | | | | 1487.6604 | |  |
|  |  |  |  |  |  | |  |  | |  | K.EIKFEFQAMDTL.- + Oxidation (M) | | | | | | | 1487.6604 | |  |
|  |  |  |  |  |  | |  |  | |  | -.MSPQTETKASVGFK.A | | | | | | | 1510.7332 | |  |
|  |  |  |  |  |  | |  |  | |  | K.ALRMSGGDHIHAGTVVGK.L | | | | | | | 1805.8870 | |  |
|  |  |  |  |  |  | |  |  | |  | K.LEGEREITLGFVDLLR.D | | | | | | | 1859.9084 | |  |
|  |  |  |  |  |  | |  |  | |  | R.EITLGFVDLLRDDFVEK.D | | | | | | | 2008.8363 | |  |
| 219 | Ribulose bisphosphate carboxylase large chain | Notothixos subaureus | [gi\|3914569](http://gps/mascot/cgi/protein_view.pl?file=../data/20131225/F038260.dat&hit=1) | 6.22/52.8 | 5.34/15 | | 268 | 21% | | 11 | R.ALRLEGLR.I | | | | | | | 927.4462 | |  |
|  |  |  |  |  |  | |  |  | |  | K.DTDILAAFR.V | | | | | | | 1021.4722 | |  |
|  |  |  |  |  |  | |  |  | |  | K.DTDILAAFR.V | | | | | | | 1021.4722 | |  |
|  |  |  |  |  |  | |  |  | |  | K.LTYYTPDYETK.D | | | | | | | 1393.5699 | |  |
|  |  |  |  |  |  | |  |  | |  | K.LTYYTPDYETK.D | | | | | | | 1393.5699 | |  |
|  |  |  |  |  |  | |  |  | |  | K.TFQGPPHGIQVER.D | | | | | | | 1465.6787 | |  |
|  |  |  |  |  |  | |  |  | |  | R.MSGGDHIHAGTVVGK.L | | | | | | | 1465.6787 | |  |
|  |  |  |  |  |  | |  |  | |  | R.DRFVFCAEAIDK.A | | | | | | | 1470.6809 | |  |
|  |  |  |  |  |  | |  |  | |  | K.EIKFEFQAMDTL.- + Oxidation (M) | | | | | | | 1487.6565 | |  |
|  |  |  |  |  |  | |  |  | |  | K.EIKFEFQAMDTL.- + Oxidation (M) | | | | | | | 1487.6565 | |  |
|  |  |  |  |  |  | |  |  | |  | R.AESSTGTWTTVWTDGLTSLDR.Y | | | | | | | 2283.991 | |  |
| 220 | ribulose-1,5-bisphosphate carboxylase/oxygenase large subunit | Houttuynia cordata | [gi\|168239](http://gps/mascot/cgi/protein_view.pl?file=../data/20131225/F038261.dat&hit=1) | 6.57/44.3 | 5.75/18 | | 233 | 20% | | 8 | K.DTDILAAFR.V | | | | | | | 1021.4820 | |    \|  \| \| --- \| |
|  |  |  |  |  |  | |  |  | |  | K.DTDILAAFR.V | | | | | | | 1021.4820 | |  |
|  |  |  |  |  |  | |  |  | |  | R.EITLGFVDLLR.D | | | | | | | 1275.6754 | |  |
|  |  |  |  |  |  | |  |  | |  | K.LTYYTPDYETK.D | | | | | | | 1393.5887 | |  |
|  |  |  |  |  |  | |  |  | |  | K.LTYYTPDYETK.D | | | | | | | 1393.5887 | |  |
|  |  |  |  |  |  | |  |  | |  | K.TFQGPPHGIQVER.D | | | | | | | 1465.6893 | |  |
|  |  |  |  |  |  | |  |  | |  | R.MSGGDHIHAGTVVGK.L | | | | | | | 1465.6893 | |  |
|  |  |  |  |  |  | |  |  | |  | R.AESSTGTWTTVWTDGLTSLDR.Y | | | | | | | 2283.9849 | |  |
| 243 | ribulose-1,5-bisphosphate carboxylase/oxygenase large subunit | Mitchella repens | [gi\|1881479](http://www.matrixscience.com/cgi/protein_view.pl?file=..%2Fdata%2F20131126%2FFTnorrceR.dat&hit=gi%7C1881479&db_idx=1&px=1&ave_thresh=48&_ignoreionsscorebelow=0&report=0&_sigthreshold=0.05&_msresflags=1025&_msresflags2=2&percolate=-1&percolate_rt=0&_minpeplen=7&sessionID=guest_guestsession) | 6.13/52.1 | 6.49/44 | | 323 | 14% | | 8 | R.AVYECLR.G | | | | | | | 910.4839 | |  |
|  |  |  |  |  |  | |  |  | |  | R.VALEACVKAR.N | | | | | | | 1116.6240 | |  |
|  |  |  |  |  |  | |  |  | |  | R.DNGLLLHIHR.A | | | | | | | 1187.7117 | |  |
|  |  |  |  |  |  | |  |  | |  | R.DITLGFVDLLR.D | | | | | | | 1261.6624 | |  |
|  |  |  |  |  |  | |  |  | |  | R.EITLGFVDLLR.D | | | | | | | 1275.7749 | |  |
|  |  |  |  |  |  | |  |  | |  | K.DDENVNSQPFMR.W | | | | | | | 1451.6686 | |  |
|  |  |  |  |  |  | |  |  | |  | K.WSPELAAACEVWK.A | | | | | | | 1546.7687 | |  |
|  |  |  |  |  |  | |  |  | |  | R.GGLDFTKDDENVNSQPFMR.W | | | | | | | 2170.0356 | |  |
| 256 | triosephosphate isomerase | Glycine max | [gi\|77540216](http://www.matrixscience.com/cgi/protein_view.pl?file=..%2Fdata%2F20131126%2FFTnorrcmR.dat&hit=gi%7C77540216&db_idx=1&px=1&ave_thresh=47&_ignoreionsscorebelow=0&report=0&_sigthreshold=0.05&_msresflags=1025&_msresflags2=2&percolate=-1&percolate_rt=0&_minpeplen=7&sessionID=guest_guestsession) | 5.87/27.4 | 6.37/44 | | 136 | 13% | | 3 | K.FFVGGNWK.C | | | | | | | 954.5062 | |    \|  \| \| --- \| |
|  |  |  |  |  |  | |  |  | |  | K.VIACIGETLEQR.E | | | | | | | 1388.7476 | |  |
| 314 | Ribulose-1,5-bisphosphate carboxylase/oxygenase large subunit | Lotononis maculata | [gi\|182411613](http://www.matrixscience.com/cgi/protein_view.pl?file=..%2Fdata%2F20131126%2FFTnorrcTt.dat&hit=gi%7C182411613&db_idx=1&px=1&ave_thresh=49&_ignoreionsscorebelow=0&report=0&_sigthreshold=0.05&_msresflags=1025&_msresflags2=2&percolate=-1&percolate_rt=0&_minpeplen=7&sessionID=guest_guestsession) | 6.16/43.2 | 6.39/56 | | 474 | 17% | | 7 | R.AVYECLR.G | | | | | | | 910.4326 | |  |
|  |  |  |  |  |  | |  |  | |  | K.DTDILAAFR.V | | | | | | | 1021.5112 | |  |
|  |  |  |  |  |  | |  |  | |  | R.DNGLLLHIHR.A | | | | | | | 1187.6458 | |  |
|  |  |  |  |  |  | |  |  | |  | R.EITLGFVDLLR.D | | | | | | | 1275.7037 | |  |
|  |  |  |  |  |  | |  |  | |  | K.SFQGPPHGIQVER.D | | | | | | | 1451.6018 | |  |
|  |  |  |  |  |  | |  |  | |  | K.TFQGPPHGIQVER.D | | | | | | | 1465.7279 | |  |
|  |  |  |  |  |  | |  |  | |  | R.GGLDFTKDDENVNSQPFMR.W | | | | | | | 2169.9475 | |  |
| 315 | Ribulose-1,5-bisphosphate carboxylase/oxygenase large subunit | Lotononis maculata | [gi\|182411613](http://www.matrixscience.com/cgi/protein_view.pl?file=..%2Fdata%2F20131126%2FFTnorrcOE.dat&hit=gi%7C182411613&db_idx=1&px=1&ave_thresh=49&_ignoreionsscorebelow=0&report=0&_sigthreshold=0.05&_msresflags=1025&_msresflags2=2&percolate=-1&percolate_rt=0&_minpeplen=7&sessionID=guest_guestsession) | 6.16/43.2 | 6.27/55 | | 411 | 17% | | 7 | R.AVYECLR.G | | | | | | | 910.4141 | |  |
|  |  |  |  |  |  | |  |  | |  | K.DTDILAAFR.V | | | | | | | 1021.4891 | |  |
|  |  |  |  |  |  | |  |  | |  | R.DNGLLLHIHR.A | | | | | | | 1187.6179 | |  |
|  |  |  |  |  |  | |  |  | |  | R.EITLGFVDLLR.D | | | | | | | 1275.6794 | |  |
|  |  |  |  |  |  | |  |  | |  | K.SFQGPPHGIQVER.D | | | | | | | 1451.5743 | |  |
|  |  |  |  |  |  | |  |  | |  | K.TFQGPPHGIQVER.D | | | | | | | 1465.7003 | |  |
|  |  |  |  |  |  | |  |  | |  | R.GGLDFTKDDENVNSQPFMR.W | | | | | | | 2169.9067 | |  |
| 318 | Ribulose-1,5-bisphosphate carboxylase/oxygenase large subunit | *Securidaca* | [gi\|194320381](http://www.matrixscience.com/cgi/protein_view.pl?file=..%2Fdata%2F20131126%2FFTnorrcOL.dat&hit=gi%7C194320381&db_idx=1&px=1&ave_thresh=49&_ignoreionsscorebelow=0&report=0&_sigthreshold=0.05&_msresflags=1025&_msresflags2=2&percolate=-1&percolate_rt=0&_minpeplen=7&sessionID=guest_guestsession) | 6.46/51.4 | 6.52/53 | | 332 | 15% | | 7 | R.AVYECLR.G | | | | | | | 910.4198 | |  |
|  |  |  |  |  |  | |  |  | |  | K.DTDILAAFR.V | | | | | | | 1021.4976 | |  |
|  |  |  |  |  |  | |  |  | |  | R.DNGLLLHIHR.A | | | | | | | 1187.6271 | |  |
|  |  |  |  |  |  | |  |  | |  | R.DITLGFVDLLR.D | | | | | | | 1261.5863 | |  |
|  |  |  |  |  |  | |  |  | |  | R.EITLGFVDLLR.D | | | | | | | 1275.6899 | |  |
|  |  |  |  |  |  | |  |  | |  | K.TFQGPPHGIQVER.D | | | | | | | 1465.7106 | |  |
|  |  |  |  |  |  | |  |  | |  | R.GGLDFTKDDENVNSQPFMR.W | | | | | | | 2169.9219 | |  |
| 321 | ribulose-1,5-bisphosphate carboxylase/oxygenase large subunit | *Duguetia confusa* | [gi\|167651221](http://www.matrixscience.com/cgi/protein_view.pl?file=..%2Fdata%2F20131126%2FFTnorrcOe.dat&hit=gi%7C167651221&db_idx=1&px=1&ave_thresh=49&_ignoreionsscorebelow=0&report=0&_sigthreshold=0.05&_msresflags=1025&_msresflags2=2&percolate=-1&percolate_rt=0&_minpeplen=7&sessionID=guest_guestsession) | 6.18/53.0 | 6.16/54 | | 302 | 10% | | 6 | R.AVYECLR.G | | | | | | | 910.4248 | |  |
|  |  |  |  |  |  | |  |  | |  | K.DTDILAAFR.V | | | | | | | 1021.5021 | |  |
|  |  |  |  |  |  | |  |  | |  | R.DNGLLLHIHR.A | | | | | | | 1187.6349 | |  |
|  |  |  |  |  |  | |  |  | |  | R.DITLGFVDLLR.D | | | | | | | 1261.5880 | |  |
|  |  |  |  |  |  | |  |  | |  | K.TFNGPPHGIQVER.D | | | | | | | 1451.5851 | |  |
|  |  |  |  |  |  | |  |  | |  | K.TFKGPPHGIQVER.D | | | | | | | 1465.7100 | |  |
| 344 | ribulose-1,5-bisphosphate carboxylase/oxygenase large subunit | *Didymosalpinx norae* | [gi\|1770216](http://www.matrixscience.com/cgi/protein_view.pl?file=..%2Fdata%2F20131126%2FFTnorrcwE.dat&hit=gi%7C1770216&db_idx=1&px=1&ave_thresh=49&_ignoreionsscorebelow=0&report=0&_sigthreshold=0.05&_msresflags=1025&_msresflags2=2&percolate=-1&percolate_rt=0&_minpeplen=7&sessionID=guest_guestsession) | 6.47/53.1 | 6.54/44 | | 514 | 16% | | 8 | R.AVYECLR.G | | | | | | | 910.4455 | |  |
|  |  |  |  |  |  | |  |  | |  | K.DTDILAAFR.V | | | | | | | 1021.5287 | |  |
|  |  |  |  |  |  | |  |  | |  | R.VALEACVKAR.N | | | | | | | 1116.5808 | |  |
|  |  |  |  |  |  | |  |  | |  | R.DPGLLLHIHR.A | | | | | | | 1170.6350 | |  |
|  |  |  |  |  |  | |  |  | |  | R.DITLGFVDLLR.D | | | | | | | 1261.6177 | |  |
|  |  |  |  |  |  | |  |  | |  | K.DDENVNSQPFMR.W | | | | | | | 1451.6265 | |  |
|  |  |  |  |  |  | |  |  | |  | K.TFQGPPHGIQVER.D | | | | | | | 1465.7522 | |  |
|  |  |  |  |  |  | |  |  | |  | R.GGLDFTKDDENVNSQPFMR.W | | | | | | | 2169.9858 | |  |
| 304 | Ribulose-1,5-bisphosphate carboxylase/oxygenase large subunit | *Lotononis maculata* | [gi\|182411613](http://www.matrixscience.com/cgi/protein_view.pl?file=..%2Fdata%2F20131126%2FFTnorrcTt.dat&hit=gi%7C182411613&db_idx=1&px=1&ave_thresh=49&_ignoreionsscorebelow=0&report=0&_sigthreshold=0.05&_msresflags=1025&_msresflags2=2&percolate=-1&percolate_rt=0&_minpeplen=7&sessionID=guest_guestsession) | 6.16/43.2 | 6.39/56 | | 474 | 17% | | 7 | R.AVYECLR.G | | | | | | | 910.4326 | |  |
|  |  |  |  |  |  | |  |  | |  | K.DTDILAAFR.V | | | | | | | 1021.5112 | |  |
|  |  |  |  |  |  | |  |  | |  | R.DNGLLLHIHR.A | | | | | | | 1187.6458 | |  |
|  |  |  |  |  |  | |  |  | |  | R.EITLGFVDLLR.D | | | | | | | 1275.7037 | |  |
|  |  |  |  |  |  | |  |  | |  | K.SFQGPPHGIQVER.D | | | | | | | 1451.6018 | |  |
|  |  |  |  |  |  | |  |  | |  | K.TFQGPPHGIQVER.D | | | | | | | 1465.7279 | |  |
|  |  |  |  |  |  | |  |  | |  | R.GGLDFTKDDENVNSQPFMR.W | | | | | | | 2169.9475 | |  |
| 362 | Ribulose bisphosphate carboxylase large chain | Fragaria x ananassa | [gi\|1352782](http://gps/mascot/cgi/protein_view.pl?file=../data/20131225/F038265.dat&hit=1) | 6.04/52.1 | 6.78/44 | | 96 | 32% | | 15 | R.AVYECLR.G | | | | | | | 910.3968 | |  |
|  |  |  |  |  |  | |  |  | |  | K.NHGMHFR.V + Oxidation (M) | | | | | | | 914.3579 | |  |
|  |  |  |  |  |  | |  |  | |  | K.DTDILAAFR.V | | | | | | | 1021.4710 | |  |
|  |  |  |  |  |  | |  |  | |  | R.VALEACVQAR.N | | | | | | | 1116.5193 | |  |
|  |  |  |  |  |  | |  |  | |  | R.QKNHGMHFR.V + Oxidation (M) | | | | | | | 1170.5720 | |  |
|  |  |  |  |  |  | |  |  | |  | R.DNGLLLHIHR.A | | | | | | | 1187.6003 | |  |
|  |  |  |  |  |  | |  |  | |  | R.DLAREGNDIIR.E | | | | | | | 1271.5115 | |  |
|  |  |  |  |  |  | |  |  | |  | R.EITLGFVDLLR.D | | | | | | | 1275.5042 | |  |
|  |  |  |  |  |  | |  |  | |  | R.EITLGFVDLLR.D | | | | | | | 1275.5042 | |  |
|  |  |  |  |  |  | |  |  | |  | K.LTYYTPDYETK.D | | | | | | | 1393.5686 | |  |
|  |  |  |  |  |  | |  |  | |  | K.TFQGPPHGIQVER.D | | | | | | | 1465.6797 | |  |
|  |  |  |  |  |  | |  |  | |  | R.MSGGDHIHAGTVVGK.L | | | | | | | 1465.6797 | |  |
|  |  |  |  |  |  | |  |  | |  | K.ALRMSGGDHIHAGTVVGK.L + Oxidation (M) | | | | | | | 1821.7805 | |  |
|  |  |  |  |  |  | |  |  | |  | R.GGLDFTKDDENVNSQPFMR.W + Oxidation (M) | | | | | | | 2185.8582 | |  |
|  |  |  |  |  |  | |  |  | |  | K.SQAETGEIKGHYLNATAGTCEDMMK.R | | | | | | | 2742.0173 | |  |
| 364 | Ribulose 1,5-bisphosphate carboxylase/oxygenase | Schima superba | [gi\|20257362](http://gps/mascot/cgi/protein_view.pl?file=../data/20131225/F038266.dat&hit=1) | 5.96/51.2 | 6.73/44 | | 249 | 35% | | 28 | R.AVYECLR.G | | | | | | | 910.3959 | |  |
|  |  |  |  |  |  | |  |  | |  | R.AVYECLR.G | | | | | | | 910.3959 | |  |
|  |  |  |  |  |  | |  |  | |  | R.AMHAVIDR.Q | | | | | | | 912.4077 | |  |
|  |  |  |  |  |  | |  |  | |  | K.NHGMHFR.V + Oxidation (M) | | | | | | | 914.3571 | |  |
|  |  |  |  |  |  | |  |  | |  | R.AMHAVIDR.Q + Oxidation (M) | | | | | | | 928.4197 | |  |
|  |  |  |  |  |  | |  |  | |  | K.AQAETGEIK.G | | | | | | | 946.4219 | |  |
|  |  |  |  |  |  | |  |  | |  | K.DTDILAAFR.V | | | | | | | 1021.4704 | |  |
|  |  |  |  |  |  | |  |  | |  | R.VALEACVQAR.N | | | | | | | 1116.5225 | |  |
|  |  |  |  |  |  | |  |  | |  | R.VALEACVQAR.N | | | | | | | 1116.5225 | |  |
|  |  |  |  |  |  | |  |  | |  | R.QKNHGMHFR.V + Oxidation (M) | | | | | | | 1170.5746 | |  |
|  |  |  |  |  |  | |  |  | |  | R.QKNHGMHFR.V + Oxidation (M) | | | | | | | 1170.5746 | |  |
|  |  |  |  |  |  | |  |  | |  | R.DNGLLLHIHR.A | | | | | | | 1187.6031 | |  |
|  |  |  |  |  |  | |  |  | |  | R.DNGLLLHIHR.A | | | | | | | 1187.6031 | |  |
|  |  |  |  |  |  | |  |  | |  | R.FLFCAEAIYK.A | | | | | | | 1261.5293 | |  |
|  |  |  |  |  |  | |  |  | |  | R.EITLGFVDLLR.D | | | | | | | 1275.6599 | |  |
|  |  |  |  |  |  | |  |  | |  | R.EITLGFVDLLR.D | | | | | | | 1275.6599 | |  |
|  |  |  |  |  |  | |  |  | |  | K.DDENVNSQPFMR.W | | | | | | | 1451.5499 | |  |
|  |  |  |  |  |  | |  |  | |  | K.EIKFEFPAMDTL.- + Oxidation (M) | | | | | | | 1456.6123 | |  |
|  |  |  |  |  |  | |  |  | |  | R.MSGGDHIHAGTVVGK.L | | | | | | | 1465.6812 | |  |
|  |  |  |  |  |  | |  |  | |  | K.DDENVNSQPFMR.W + Oxidation (M) | | | | | | | 1467.6017 | |  |
|  |  |  |  |  |  | |  |  | |  | K.WSPELAAACEVWK.E | | | | | | | 1546.6399 | |  |
|  |  |  |  |  |  | |  |  | |  | K.GHYLNATAGTCEEMIK.R | | | | | | | 1794.7351 | |  |
|  |  |  |  |  |  | |  |  | |  | K.GHYLNATAGTCEEMIK.R + Oxidation (M) | | | | | | | 1810.7037 | |  |
|  |  |  |  |  |  | |  |  | |  | K.ALRMSGGDHIHAGTVVGK.L + Oxidation (M) | | | | | | | 1821.7781 | |  |
|  |  |  |  |  |  | |  |  | |  | K.GHYLNATAGTCEEMIKR.A | | | | | | | 1950.8130 | |  |
|  |  |  |  |  |  | |  |  | |  | K.GHYLNATAGTCEEMIKR.A + Oxidation (M) | | | | | | | 1966.7987 | |  |
|  |  |  |  |  |  | |  |  | |  | R.GGLDFTKDDENVNSQPFMR.W | | | | | | | 2169.8696 | |  |
|  |  |  |  |  |  | |  |  | |  | R.GGLDFTKDDENVNSQPFMR.W + Oxidation (M) | | | | | | | 2185.8481 | |  |
| 366 | fructose-bisphosphate aldolase | *Glycine max* | [gi\|356508188](http://www.matrixscience.com/cgi/protein_view.pl?file=..%2Fdata%2F20131126%2FFTnorrcwe.dat&hit=gi%7C356508188&db_idx=1&px=1&ave_thresh=47&_ignoreionsscorebelow=0&report=0&_sigthreshold=0.05&_msresflags=1025&_msresflags2=2&percolate=-1&percolate_rt=0&_minpeplen=7&sessionID=guest_guestsession) | 6.38/42.8 | 6.56/37 | | 121 | 8% | | 3 | K.AAQDALLFR.A | | | | | | | 1004.5364 | |    \|  \| \| --- \| |
|  |  |  |  |  |  | |  |  | |  | R.SAAYYQQGAR.F | | | | | | | 1114.5139 | |  |
|  |  |  |  |  |  | |  |  | |  | R.LASIGLENTEANR.Q | | | | | | | 1387.7008 | |  |
| 368 | unnamed protein product | Vitis vinifera | [gi\|296086099](http://gps/mascot/cgi/protein_view.pl?file=../data/20131225/F038268.dat&hit=1) | 5.95/42.8 | 5.05/37 | | 114 | 13% | | 8 | K.LFSPGNLR.A | | | | | | | 903.4614 | |  |
|  |  |  |  |  |  | |  |  | |  | K.LFSPGNLR.A | | | | | | | 903.4614 | |  |
|  |  |  |  |  |  | |  |  | |  | K.LINYYVR.E | | | | | | | 940.4675 | |  |
|  |  |  |  |  |  | |  |  | |  | R.FEETLYGSSR.L | | | | | | | 1188.4979 | |  |
|  |  |  |  |  |  | |  |  | |  | R.FEETLYGSSR.L | | | | | | | 1188.4979 | |  |
|  |  |  |  |  |  | |  |  | |  | R.LLFEVAPLGFLIEK.A | | | | | | | 1588.8458 | |  |
|  |  |  |  |  |  | |  |  | |  | R.LLFEVAPLGFLIEK.A | | | | | | | 1588.8458 | |  |
|  |  |  |  |  |  | |  |  | |  | R.YTGGMVPDVNQIIVK.E + Oxidation (M) | | | | | | | 1649.7623 | |  |
| 446 | Ribulose bisphosphate carboxylase large chain | Zamia integrifolia | [gi\|131899](http://www.matrixscience.com/cgi/protein_view.pl?file=..%2Fdata%2F20131128%2FFTnooGTwR.dat&hit=gi%7C131899&db_idx=1&px=1&ave_thresh=48&_ignoreionsscorebelow=0&report=10&_sigthreshold=0.05&_msresflags=1025&_msresflags2=2&percolate=-1&percolate_rt=0&_minpeplen=7&sessionID=guest_guestsession) | 6.13/52.2 | 6.24/20 | | 233 | 7% | | 3 | K.DTDILAAFR.V | | | | | | | 1021.5294 | |  |
| 448 | Ribulose bisphosphate carboxylase large chain | *Zamia integrifolia* | [gi\|131925](http://www.matrixscience.com/cgi/protein_view.pl?file=..%2Fdata%2F20131128%2FFTnooGTam.dat&hit=gi%7C131925&db_idx=1&px=1&ave_thresh=47&_ignoreionsscorebelow=0&report=10&_sigthreshold=0.05&_msresflags=1025&_msresflags2=2&percolate=-1&percolate_rt=0&_minpeplen=7&sessionID=guest_guestsession) | 6.44/52.2 | 5.41/18 | | 230 | 7% | | 3 | K.DTDILAAFR.V | | | | | | | 1021.5295 | |    \|  \| \| --- \| |
|  |  |  |  |  |  | |  |  | |  | K.LTYYTPDYETK.D | | | | | | | 1393.6584 | |  |
|  |  |  |  |  |  | |  |  | |  | K.TFQGPPHGIQVER.D | | | | | | | 1465.7550 | |  |
| 454 | Ribulose bisphosphate carboxylase large chain | *Psyllocarpus laricoides* | [gi\|131981](http://www.matrixscience.com/cgi/protein_view.pl?file=..%2Fdata%2F20131128%2FFTnooGTnT.dat&hit=gi%7C131981&db_idx=1&px=1&ave_thresh=47&_ignoreionsscorebelow=0&report=10&_sigthreshold=0.05&_msresflags=1025&_msresflags2=2&percolate=-1&percolate_rt=0&_minpeplen=7&sessionID=guest_guestsession) | 6.10/51.9 | 5.84/15 | | 202 | 7% | | 3 | R.VALEACVKAR.N | | | | | | | 1116.5747 | |  |
|  |  |  |  |  |  | |  |  | |  | R.EITLGFVDLLR.D | | | | | | | 1275.7161 | |  |
|  |  |  |  |  |  | |  |  | |  | K.WSPELAAACEVWK.E | | | | | | | 1546.7189 | |  |
| 455 | Ribulose-1,5-bisphosphate carboxylase/oxygenase large subunit | *Ipomoea coccinea* | [gi\|348924](http://www.matrixscience.com/cgi/protein_view.pl?file=..%2Fdata%2F20131128%2FFTnooGTth.dat&hit=gi%7C348924&db_idx=1&px=1&ave_thresh=47&_ignoreionsscorebelow=0&report=10&_sigthreshold=0.05&_msresflags=1025&_msresflags2=2&percolate=-1&percolate_rt=0&_minpeplen=7&sessionID=guest_guestsession) | 6.57/49.7 | 5.28/14 | | 236 | 11% | | 4 | R.VALEACVKAR.N | | | | | | | 1116.5717 | |  |
|  |  |  |  |  |  | |  |  | |  | R.EITLGFVDLLR.D | | | | | | | 1275.7151 | |  |
|  |  |  |  |  |  | |  |  | |  | R.LSGGDHIHAGTVVGK.L | | | | | | | 1447.7422 | |  |
|  |  |  |  |  |  | |  |  | |  | K.WSPELAAACEVWK.E | | | | | | | 1546.7147 | |  |
| 458 | Ribulose bisphosphate carboxylase large chain | Bulnesia arborea | [gi\|17367461](http://gps/mascot/cgi/protein_view.pl?file=../data/20131225/F038269.dat&hit=1) | 6.18/52.0 | 5.05/14 | | 382 | 17% | | 18 | R.DDFVEKDR.S | | | | | | | 1023.4080 | |  |
|  |  |  |  |  |  | |  |  | |  | R.DDFVEKDR.S | | | | | | | 1023.4080 | |  |
|  |  |  |  |  |  | |  |  | |  | K.FEFPAMDTL.- | | | | | | | 1070.4081 | |  |
|  |  |  |  |  |  | |  |  | |  | R.VALEACVQAR.N | | | | | | | 1116.5116 | |  |
|  |  |  |  |  |  | |  |  | |  | R.VALEACVQAR.N | | | | | | | 1116.5116 | |  |
|  |  |  |  |  |  | |  |  | |  | R.DLAREGNTIIR.E | | | | | | | 1257.6466 | |  |
|  |  |  |  |  |  | |  |  | |  | R.DLAREGNTIIR.E | | | | | | | 1257.6466 | |  |
|  |  |  |  |  |  | |  |  | |  | R.EITLGFVDLLR.D | | | | | | | 1275.6533 | |  |
|  |  |  |  |  |  | |  |  | |  | R.EITLGFVDLLR.D | | | | | | | 1275.6533 | |  |
|  |  |  |  |  |  | |  |  | |  | K.EIKFEFPAMDTL.- | | | | | | | 1440.6129 | |  |
|  |  |  |  |  |  | |  |  | |  | R.LSGGDHIHAGTVVGK.L | | | | | | | 1447.6761 | |  |
|  |  |  |  |  |  | |  |  | |  | R.LSGGDHIHAGTVVGK.L | | | | | | | 1447.6761 | |  |
|  |  |  |  |  |  | |  |  | |  | K.EIKFEFPAMDTL.- + Oxidation (M) | | | | | | | 1456.6056 | |  |
|  |  |  |  |  |  | |  |  | |  | K.EIKFEFPAMDTL.- + Oxidation (M) | | | | | | | 1456.6056 | |  |
|  |  |  |  |  |  | |  |  | |  | K.WSPELAAACEVWK.E | | | | | | | 1546.6389 | |  |
|  |  |  |  |  |  | |  |  | |  | K.WSPELAAACEVWK.E | | | | | | | 1546.6389 | |  |
|  |  |  |  |  |  | |  |  | |  | R.EITLGFVDLLRDDFVEK.D | | | | | | | 2008.9414 | |  |
|  |  |  |  |  |  | |  |  | |  | R.EITLGFVDLLRDDFVEK.D | | | | | | | 2008.9414 | |  |
| 459 | Ribulose bisphosphate carboxylase large chain | Bulnesia arborea | [gi\|17367461](http://gps/mascot/cgi/protein_view.pl?file=../data/20131225/F038269.dat&hit=1) | 6.18/52.0 | 4.70/14 | | 400 | 17% | | 17 | R.DDFVEKDR.S | | | | | | | 1023.4041 | |  |
|  |  |  |  |  |  | |  |  | |  | R.DDFVEKDR.S | | | | | | | 1023.4041 | |  |
|  |  |  |  |  |  | |  |  | |  | R.VALEACVQAR.N | | | | | | | 1116.5073 | |  |
|  |  |  |  |  |  | |  |  | |  | R.VALEACVQAR.N | | | | | | | 1116.5073 | |  |
|  |  |  |  |  |  | |  |  | |  | R.DLAREGNTIIR.E | | | | | | | 1257.6416 | |  |
|  |  |  |  |  |  | |  |  | |  | R.DLAREGNTIIR.E | | | | | | | 1257.6416 | |  |
|  |  |  |  |  |  | |  |  | |  | R.EITLGFVDLLR.D | | | | | | | 1275.6453 | |  |
|  |  |  |  |  |  | |  |  | |  | R.EITLGFVDLLR.D | | | | | | | 1275.6453 | |  |
|  |  |  |  |  |  | |  |  | |  | K.EIKFEFPAMDTL.- | | | | | | | 1440.6029 | |  |
|  |  |  |  |  |  | |  |  | |  | K.EIKFEFPAMDTL.- | | | | | | | 1440.6029 | |  |
|  |  |  |  |  |  | |  |  | |  | R.LSGGDHIHAGTVVGK.L | | | | | | | 1447.6716 | |  |
|  |  |  |  |  |  | |  |  | |  | K.EIKFEFPAMDTL.- + Oxidation (M) | | | | | | | 1456.5981 | |  |
|  |  |  |  |  |  | |  |  | |  | K.EIKFEFPAMDTL.- + Oxidation (M) | | | | | | | 1456.5981 | |  |
|  |  |  |  |  |  | |  |  | |  | K.WSPELAAACEVWK.E | | | | | | | 1546.6282 | |  |
|  |  |  |  |  |  | |  |  | |  | K.WSPELAAACEVWK.E | | | | | | | 1546.6282 | |  |
|  |  |  |  |  |  | |  |  | |  | R.EITLGFVDLLRDDFVEK.D | | | | | | | 2008.9343 | |  |
|  |  |  |  |  |  | |  |  | |  | R.EITLGFVDLLRDDFVEK.D | | | | | | | 2008.9343 | |  |
| 480 | Ribulose bisphosphate carboxylase large chain | Bulnesia arborea | [gi\|17367461](http://gps/mascot/cgi/protein_view.pl?file=../data/20131225/F038269.dat&hit=1) | 6.18/52.0 | 4.81/14 | | 417 | 19% | | 21 | R.DDFVEKDR.S | | | | | | | 1023.4131 | |  |
|  |  |  |  |  |  | |  |  | |  | R.DDFVEKDR.S | | | | | | | 1023.4131 | |  |
|  |  |  |  |  |  | |  |  | |  | K.FEFPAMDTL.- | | | | | | | 1070.4166 | |  |
|  |  |  |  |  |  | |  |  | |  | K.FEFPAMDTL.- + Oxidation (M) | | | | | | | 1086.3993 | |  |
|  |  |  |  |  |  | |  |  | |  | R.VALEACVQAR.N | | | | | | | 1116.5117 | |  |
|  |  |  |  |  |  | |  |  | |  | R.VALEACVQAR.N | | | | | | | 1116.5117 | |  |
|  |  |  |  |  |  | |  |  | |  | R.QKNHGMHFR.V | | | | | | | 1154.4789 | |  |
|  |  |  |  |  |  | |  |  | |  | R.QKNHGMHFR.V + Oxidation (M) | | | | | | | 1170.4379 | |  |
|  |  |  |  |  |  | |  |  | |  | R.DLAREGNTIIR.E | | | | | | | 1257.6526 | |  |
|  |  |  |  |  |  | |  |  | |  | R.DLAREGNTIIR.E | | | | | | | 1257.6526 | |  |
|  |  |  |  |  |  | |  |  | |  | R.EITLGFVDLLR.D | | | | | | | 1275.6472 | |  |
|  |  |  |  |  |  | |  |  | |  | R.EITLGFVDLLR.D | | | | | | | 1275.6472 | |  |
|  |  |  |  |  |  | |  |  | |  | K.EIKFEFPAMDTL.- | | | | | | | 1440.6096 | |  |
|  |  |  |  |  |  | |  |  | |  | R.LSGGDHIHAGTVVGK.L | | | | | | | 1447.6869 | |  |
|  |  |  |  |  |  | |  |  | |  | R.LSGGDHIHAGTVVGK.L | | | | | | | 1447.6869 | |  |
|  |  |  |  |  |  | |  |  | |  | K.EIKFEFPAMDTL.- + Oxidation (M) | | | | | | | 1456.6066 | |  |
|  |  |  |  |  |  | |  |  | |  | K.EIKFEFPAMDTL.- + Oxidation (M) | | | | | | | 1456.6066 | |  |
|  |  |  |  |  |  | |  |  | |  | K.WSPELAAACEVWK.E | | | | | | | 1546.6351 | |  |
|  |  |  |  |  |  | |  |  | |  | K.WSPELAAACEVWK.E | | | | | | | 1546.6351 | |  |
|  |  |  |  |  |  | |  |  | |  | R.EITLGFVDLLRDDFVEK.D | | | | | | | 2008.9412 | |  |
|  |  |  |  |  |  | |  |  | |  | R.EITLGFVDLLRDDFVEK.D | | | | | | | 2008.9412 | |  |
| 500 | Ribulose bisphosphate carboxylase large chain | Bulnesia arborea | [gi\|17367461](http://gps/mascot/cgi/protein_view.pl?file=../data/20131225/F038269.dat&hit=1) | 6.18/52.0 | 4.98/44 | | 379 | 17% | | 18 | R.DDFVEKDR.S | | | | | | | 1023.3984 | |  |
|  |  |  |  |  |  | |  |  | |  | R.DDFVEKDR.S | | | | | | | 1023.3984 | |  |
|  |  |  |  |  |  | |  |  | |  | K.FEFPAMDTL.- + Oxidation (M) | | | | | | | 1086.3885 | |  |
|  |  |  |  |  |  | |  |  | |  | R.VALEACVQAR.N | | | | | | | 1116.5028 | |  |
|  |  |  |  |  |  | |  |  | |  | R.VALEACVQAR.N | | | | | | | 1116.5028 | |  |
|  |  |  |  |  |  | |  |  | |  | R.DLAREGNTIIR.E | | | | | | | 1257.6349 | |  |
|  |  |  |  |  |  | |  |  | |  | R.DLAREGNTIIR.E | | | | | | | 1257.6349 | |  |
|  |  |  |  |  |  | |  |  | |  | R.EITLGFVDLLR.D | | | | | | | 1275.6396 | |  |
|  |  |  |  |  |  | |  |  | |  | R.EITLGFVDLLR.D | | | | | | | 1275.6396 | |  |
|  |  |  |  |  |  | |  |  | |  | K.EIKFEFPAMDTL.- | | | | | | | 1440.6001 | |  |
|  |  |  |  |  |  | |  |  | |  | R.LSGGDHIHAGTVVGK.L | | | | | | | 1447.6665 | |  |
|  |  |  |  |  |  | |  |  | |  | R.LSGGDHIHAGTVVGK.L | | | | | | | 1447.6665 | |  |
|  |  |  |  |  |  | |  |  | |  | K.EIKFEFPAMDTL.- + Oxidation (M) | | | | | | | 1456.5916 | |  |
|  |  |  |  |  |  | |  |  | |  | K.EIKFEFPAMDTL.- + Oxidation (M) | | | | | | | 1456.5916 | |  |
|  |  |  |  |  |  | |  |  | |  | K.WSPELAAACEVWK.E | | | | | | | 1546.6238 | |  |
|  |  |  |  |  |  | |  |  | |  | K.WSPELAAACEVWK.E | | | | | | | 1546.6238 | |  |
|  |  |  |  |  |  | |  |  | |  | R.EITLGFVDLLRDDFVEK.D | | | | | | | 2008.9224 | |  |
|  |  |  |  |  |  | |  |  | |  | R.EITLGFVDLLRDDFVEK.D | | | | | | | 2008.9224 | |  |
| **Photosynthesis Electron Transfer** | | | | | | | | | | | | | | | | | | | | |
| 123 | ATP synthase CF1 beta subunit | | | | | Chloranthus spicatus | | | [gi\|146744195](http://gps/mascot/cgi/protein_view.pl?file=../data/20131225/F038254.dat&hit=1) | | | 5.16/53.6 |  | 360 | 54% | 33 | K.IGLFGGAGVGK.T | | 975.5128 |    \|  \| \| --- \| |
|  |  | | | | |  | | |  | | |  |  |  |  |  | K.LSIFETGIK.V | | 1007.5232 |  |
|  |  | | | | |  | | |  | | |  |  |  |  |  | K.YVGLAETIR.G | | 1021.4680 |  |
|  |  | | | | |  | | |  | | |  |  |  |  |  | K.VVDLLAPYR.R | | 1045.5518 |  |
|  |  | | | | |  | | |  | | |  |  |  |  |  | K.VVDLLAPYR.R | | 1045.5518 |  |
|  |  | | | | |  | | |  | | |  |  |  |  |  | R.SAPAFIQLDTK.L | | 1190.5785 |  |
|  |  | | | | |  | | |  | | |  |  |  |  |  | K.VVDLLAPYRR.G | | 1201.6493 |  |
|  |  | | | | |  | | |  | | |  |  |  |  |  | R.AVAMSATDGLMR.G + 2 Oxidation (M) | | 1254.5210 |  |
|  |  | | | | |  | | |  | | |  |  |  |  |  | K.AMNLEVESKLK.K + Oxidation (M) | | 1277.6274 |  |
|  |  | | | | |  | | |  | | |  |  |  |  |  | K.AHGGVSVFGGVGER.T | | 1328.6097 |  |
|  |  | | | | |  | | |  | | |  |  |  |  |  | K.AHGGVSVFGGVGER.T | | 1328.6097 |  |
|  |  | | | | |  | | |  | | |  |  |  |  |  | R.IVGEEHYETAQR.V | | 1431.6217 |  |
|  |  | | | | |  | | |  | | |  |  |  |  |  | R.FVQAGSEVSALLGR.M | | 1433.6973 |  |
|  |  | | | | |  | | |  | | |  |  |  |  |  | R.FVQAGSEVSALLGR.M | | 1433.6973 |  |
|  |  | | | | |  | | |  | | |  |  |  |  |  | R.VGLTALTMAEYFR.D | | 1471.6982 |  |
|  |  | | | | |  | | |  | | |  |  |  |  |  | R.VGLTALTMAEYFR.D + Oxidation (M) | | 1487.6868 |  |
|  |  | | | | |  | | |  | | |  |  |  |  |  | R.VGLTALTMAEYFR.D + Oxidation (M) | | 1487.6868 |  |
|  |  | | | | |  | | |  | | |  |  |  |  |  | R.TREGNDLYMEMK.E + 2 Oxidation (M) | | 1518.5863 |  |
|  |  | | | | |  | | |  | | |  |  |  |  |  | K.VALVYGQMNEPPGAR.M | | 1601.7408 |  |
|  |  | | | | |  | | |  | | |  |  |  |  |  | K.VALVYGQMNEPPGAR.M + Oxidation (M) | | 1617.7291 |  |
|  |  | | | | |  | | |  | | |  |  |  |  |  | K.VALVYGQMNEPPGAR.M + Oxidation (M) | | 1617.7291 |  |
|  |  | | | | |  | | |  | | |  |  |  |  |  | R.IAQIIGPVLDVAFPPGK.M | | 1734.9164 |  |
|  |  | | | | |  | | |  | | |  |  |  |  |  | R.DVNEQDVLLFIDNIFR.F | | 1949.9119 |  |
|  |  | | | | |  | | |  | | |  |  |  |  |  | K.GIYPAVDPLDSTSTMLQPR.I | | 2060.9531 |  |
|  |  | | | | |  | | |  | | |  |  |  |  |  | K.GIYPAVDPLDSTSTMLQPR.I + Oxidation (M) | | 2076.9263 |  |
|  |  | | | | |  | | |  | | |  |  |  |  |  | K.GIYPAVDPLDSTSTMLQPR.I + Oxidation (M) | | 2076.9263 |  |
|  |  | | | | |  | | |  | | |  |  |  |  |  | R.GMEVIDTGAPLSVPVGGATLGR.I + Oxidation (M) | | 2112.9863 |  |
|  |  | | | | |  | | |  | | |  |  |  |  |  | R.GMEVIDTGAPLSVPVGGATLGR.I + Oxidation (M) | | 2112.9863 |  |
|  |  | | | | |  | | |  | | |  |  |  |  |  | R.MPSAVGYQPTLSTEMGSLQER.I + Oxidation (M) | | 2297.9849 |  |
|  |  | | | | |  | | |  | | |  |  |  |  |  | R.MPSAVGYQPTLSTEMGSLQER.I + 2 Oxidation (M) | | 2313.9536 |  |
|  |  | | | | |  | | |  | | |  |  |  |  |  | R.DTVGQQINVTCEVQQLLGNNR.V | | 2386.0693 |  |
|  |  | | | | |  | | |  | | |  |  |  |  |  | R.DTVGQQINVTCEVQQLLGNNR.V | | 2386.0693 |  |
| 213 | ferredoxin--NADP reductase | | | | | *Glycine max* | | | [gi\|356502850](http://www.matrixscience.com/cgi/protein_view.pl?file=..%2Fdata%2F20131126%2FFTnorrcme.dat&hit=gi%7C356502850&db_idx=1&px=1&ave_thresh=46&_ignoreionsscorebelow=0&report=0&_sigthreshold=0.05&_msresflags=1025&_msresflags2=2&percolate=-1&percolate_rt=0&_minpeplen=7&sessionID=guest_guestsession) | | | 8.38/40.6 | 6.61/44 | 167 | 15% | 4 | K.KAEQWNVEVY.- | | 1265.6212 |    \|  \| \| --- \| |
|  |  | | | | |  | | |  | | |  |  |  |  |  | K.DGIDWTEYKR.Q | | 1282.6179 |  |
|  |  | | | | |  | | |  | | |  |  |  |  |  | R.LYSIASSAIGDFGDSK.T | | 1630.8033 |  |
|  |  | | | | |  | | |  | | |  |  |  |  |  | K.DPNATIIMLGTGTGIAPFR.S | | 1945.0370 |  |
| 258 | Chlorophyll a-b binding protein 3C-like protein | | | | | *Medicago truncatula* | | | [gi\|357497757](http://www.matrixscience.com/cgi/protein_view.pl?file=..%2Fdata%2F20131126%2FFTnorrcTT.dat&hit=gi%7C357497757&db_idx=1&px=1&ave_thresh=47&_ignoreionsscorebelow=0&report=0&_sigthreshold=0.05&_msresflags=1025&_msresflags2=2&percolate=-1&percolate_rt=0&_minpeplen=7&sessionID=guest_guestsession) | | | 5.53/24.9 | 4.96/44 | 113 | 9% | 3 | R.ELEVIHSR.W | | 982.5612 |  |
|  |  | | | | |  | | |  | | |  |  |  |  |  | K.VSSGSPWYGPDR.V | | 1307.6272 |  |
|  |  | | | | |  | | |  | | |  |  |  |  |  | K.KVSSGSPWYGPDR.V | | 1435.7234 |  |
| 278 | ATP synthase beta subunit | | | | | *Dryopteris filix-mas* | | | [gi\|169260987](http://gps/mascot/cgi/protein_view.pl?file=../data/20131128/F036070.dat&hit=5) | | | 5.22/33.3 | 5.84/73 | 42 | 28% | 8 | K.TVLITESINNIAK.A | | 1415.7214 |  |
|  |  | | | | |  | | |  | | |  |  |  |  |  | R.VGSTALTMAEYFR.D + Oxidation (M) | | 1461.8480 |  |
|  |  | | | | |  | | |  | | |  |  |  |  |  | R.TREGNDLYMEMK.E + Oxidation (M) | | 1502.8260 |  |
|  |  | | | | |  | | |  | | |  |  |  |  |  | R.EGNDLYMEMKESK.V + Oxidation (M) | | 1589.7866 |  |
|  |  | | | | |  | | |  | | |  |  |  |  |  | R.MRVGSTALTMAEYFR.D + Oxidation (M) | | 1748.7386 |  |
|  |  | | | | |  | | |  | | |  |  |  |  |  | K.VALVYGQMNEPPGARMR.V | | 1888.9578 |  |
|  |  | | | | |  | | |  | | |  |  |  |  |  | R.VGSTALTMAEYFRDVNK.Q | | 1901.9224 |  |
|  |  | | | | |  | | |  | | |  |  |  |  |  | R.DVNKQDVPLFIDNIFR.F | | 1932.9288 |  |
| 308 | ATP synthase CF1 alpha subunit | | | | | *Oryza sativa Japonica Group* | | | [gi\|11466784](http://www.matrixscience.com/cgi/protein_view.pl?file=..%2Fdata%2F20131126%2FFTnorrcOO.dat&hit=gi%7C11466784&db_idx=1&px=1&ave_thresh=46&_ignoreionsscorebelow=0&report=0&_sigthreshold=0.05&_msresflags=1025&_msresflags2=2&percolate=-1&percolate_rt=0&_minpeplen=7&sessionID=guest_guestsession) | | | 5.95/55.7 | 5.10/60 | 174 | 11% | 4 | R.LIESPAPGIISR.R | | 1252.7003 |  |
|  |  | | | | |  | | |  | | |  |  |  |  |  | R.IAQIPVSEAYLGR.V | | 1416.7561 |  |
|  |  | | | | |  | | |  | | |  |  |  |  |  | R.EAYPGDVFYLHSR.L | | 1553.7063 |  |
|  |  | | | | |  | | |  | | |  |  |  |  |  | R.SVYEPLQTGLIAIDSMIPIGR.G | | 2273.1709 |  |
| 408 | Chlorophyll a-b binding protein 3C-like protein | | | | | *Medicago truncatula* | | | [gi\|357497757](http://www.matrixscience.com/cgi/protein_view.pl?file=..%2Fdata%2F20131126%2FFTnorrcaO.dat&hit=gi%7C357497757&db_idx=1&px=1&ave_thresh=47&_ignoreionsscorebelow=0&report=0&_sigthreshold=0.05&_msresflags=1025&_msresflags2=2&percolate=-1&percolate_rt=0&_minpeplen=7&sessionID=guest_guestsession) | | | 5.53/24.9 | 5.06/26 | 77 | 5% | 2 | K.VSSGSPWYGPDR.V | | 1307.5946 |  |
|  |  | | | | |  | | |  | | |  |  |  |  |  | K.KVSSGSPWYGPDR.V | | 1435.6875 |  |
| 444 | ATP synthase delta chain | | | | | *Glycine max* | | | [gi\|356514035](http://www.matrixscience.com/cgi/protein_view.pl?file=..%2Fdata%2F20131128%2FFTnooGTwL.dat&hit=gi%7C356514035&db_idx=1&px=1&ave_thresh=47&_ignoreionsscorebelow=0&report=10&_sigthreshold=0.05&_msresflags=1025&_msresflags2=2&percolate=-1&percolate_rt=0&_minpeplen=7&sessionID=guest_guestsession) | | | 6.92/26.8 | 4.83/20 | 63 | 4% | 1 | R.NFLYILVDAQR.I | | 1351.7407 |  |
|    \| **Regulation/Defense** \| \| --- \| | | | | | | | | | | | | | | | | | | | | |
| 80 | leaf rust resistance protein | | | | | *Triticum dicoccoides* | | | gi\|305691103 | | | 5.68/92.6 | 6.06/68 | 43 | 31% | 1 | YLYELVK | | 927.5358 |    \|  \| \| --- \| |
| 279 | leaf rust resistance protein Lr10 | | | | | *Triticum dicoccoides* | | | gi\|305691147 | | | 9.44/44.9 | 5.93/73 | 53 | 30% | 13 | R.GLVKYDSLTK.S | |  |  |
|  |  | | | | |  | | |  | | |  |  |  |  |  | K.YIADASSPNVGVTK.C | |  |  |
|  |  | | | | |  | | |  | | |  |  |  |  |  | K.RENDTMIQDIWR.L | |  |  |
|  |  | | | | |  | | |  | | |  |  |  |  |  | K.SIFHLAFVKATDYK.L | |  |  |
|  |  | | | | |  | | |  | | |  |  |  |  |  | K.SIFHLAFVKATDYK.L | |  |  |
|  |  | | | | |  | | |  | | |  |  |  |  |  | K.DLVEPSTPVAVCKNK.K | |  |  |
|  |  | | | | |  | | |  | | |  |  |  |  |  | R.VNAWRYLACTPSHR.I | |  |  |
|  |  | | | | |  | | |  | | |  |  |  |  |  | K.ISESCDGLFCIHSPK.S | |  |  |
|  |  | | | | |  | | |  | | |  |  |  |  |  | K.ISESCDGLFCIHSPK.S | |  |  |
|  |  | | | | |  | | |  | | |  |  |  |  |  | M.MEAEQQVVMASSSSLR.K | |  |  |
|  |  | | | | |  | | |  | | |  |  |  |  |  | M.MEAEQQVVMASSSSLRK.R | |  |  |
|  |  | | | | |  | | |  | | |  |  |  |  |  | M.MEAEQQVVMASSSSLRK.R | |  |  |
|  |  | | | | |  | | |  | | |  |  |  |  |  | R.WLRLLPPAGFQILIHK.F | |  |  |
| 413 | L-ascorbate peroxidase | | | | | *Cicer arietinum* | | | [gi\|502145236](http://www.matrixscience.com/cgi/protein_view.pl?file=..%2Fdata%2F20131126%2FFTnorrcaS.dat&hit=gi%7C502145236&db_idx=1&px=1&ave_thresh=46&_ignoreionsscorebelow=0&report=0&_sigthreshold=0.05&_msresflags=1025&_msresflags2=2&percolate=-1&percolate_rt=0&_minpeplen=7&sessionID=guest_guestsession) | | | 5.65/27.2 | 5.99/25 | 163 | 12% | 2 | R.LAWHSAGTFDVK.T | | 1331.6785 |  |
| 422 | lectin | | | | | *Astragalus falcatus* | | | [gi\|3819113](http://www.matrixscience.com/cgi/protein_view.pl?file=..%2Fdata%2F20131126%2FFTnorrcaL.dat&hit=gi%7C3819113&db_idx=1&px=1&ave_thresh=46&_ignoreionsscorebelow=0&report=0&_sigthreshold=0.05&_msresflags=1025&_msresflags2=2&percolate=-1&percolate_rt=0&_minpeplen=7&sessionID=guest_guestsession) | | | 3.85/9.85 | 5.87/24 | 66 | 8% | 1 | R.EVLPEWVR.I | | 1027.5521 |  |
| 423 | ferritin-3 | | | | | *Glycine max* | | | [gi\|29839257](http://www.matrixscience.com/cgi/protein_view.pl?file=..%2Fdata%2F20131128%2FFTnooGTTR.dat&hit=gi%7C29839257&db_idx=1&px=1&ave_thresh=46&_ignoreionsscorebelow=0&report=10&_sigthreshold=0.05&_msresflags=1025&_msresflags2=2&percolate=-1&percolate_rt=0&_minpeplen=7&sessionID=guest_guestsession) | | | 5.54/28.6 | 5.29/24 | 103 | 7% | 2 | K.IAEYVTQLR.L | | 1092.5922 |  |
|  |  | | | | |  | | |  | | |  |  |  |  |  | K.GHGVWHFDQR.L | | 1238.5702 |  |
| **Chaperone** | | | | | | | | | | | | | | | | | | | | |
| 45 | chaperone protein ClpC, chloroplastic-like isoform 1 | | | | | *Glycine max* | | | [gi\|356508861](http://www.matrixscience.com/cgi/protein_view.pl?file=..%2Fdata%2F20131125%2FFTnornHeh.dat&hit=gi%7C356508861&db_idx=1&px=1&ave_thresh=46&_ignoreionsscorebelow=0&report=10&_sigthreshold=0.05&_msresflags=1025&_msresflags2=2&percolate=-1&percolate_rt=0&_minpeplen=7&sessionID=guest_guestsession) | | | 6.16/102 | 5.50/90 | 267 | 11% | 7 | R.LQHAQLPEEAR.E | | 1291.7068 |  |
|  |  | | | | |  | | |  | | |  |  |  |  |  | R.VLENLGADPTNIR.T | | 1411.7841 |  |
|  |  | | | | |  | | |  | | |  |  |  |  |  | R.GSGFVAVEIPFTPR.A | | 1476.8157 |  |
|  |  | | | | |  | | |  | | |  |  |  |  |  | K.ALAAYYFGSEEAMIR.L | | 1691.8453 |  |
|  |  | | | | |  | | |  | | |  |  |  |  |  | R.GELQCIGATTLDEYRK.H | | 1853.9437 |  |
|  |  | | | | |  | | |  | | |  |  |  |  |  | K.NPNRPIASFIFSGPTGVGK.S | | 1959.0874 |  |
|  |  | | | | |  | | |  | | |  |  |  |  |  | K.LIGSPPGYVGYTEGGQLTEAVR.R | | 2264.2012 |  |
| 47 | heat shock protein, putative | | | | | *Ricinus communis* | | | [gi\|255582806](http://www.matrixscience.com/cgi/protein_view.pl?file=..%2Fdata%2F20131126%2FFTnorrcEt.dat&hit=gi%7C255582806&db_idx=1&px=1&ave_thresh=47&_ignoreionsscorebelow=0&report=0&_sigthreshold=0.05&_msresflags=1025&_msresflags2=2&percolate=-1&percolate_rt=0&_minpeplen=7&sessionID=guest_guestsession) | | | 5.01/80.2 | 4.88/89 | 267 | 11% | 7 | R.APFDLFDTR.K | | 1081.5647 |  |
|  |  | | | | |  | | |  | | |  |  |  |  |  | K.RAPFDLFDTR.K | | 1237.6659 |  |
|  |  | | | | |  | | |  | | |  |  |  |  |  | K.ADLVNNLGTIAR.S | | 1256.7329 |  |
|  |  | | | | |  | | |  | | |  |  |  |  |  | K.EDQLEYLEER.R | | 1323.6490 |  |
|  |  | | | | |  | | |  | | |  |  |  |  |  | K.GIVDSEDLPLNISR.E | | 1527.8474 |  |
|  |  | | | | |  | | |  | | |  |  |  |  |  | K.LDAQPELFIHIIPDK.T | | 1748.9943 |  |
|  |  | | | | |  | | |  | | |  |  |  |  |  | K.HNDDEQYVWESQAGGSFTVTR.D | | 2426.1172 |  |
| 280 | Chlorophyllase-2, chloroplast precursor, putative | | | | | *Ricinus communis* | | | [gi\|223543710](http://gps/mascot/cgi/protein_view.pl?file=../data/20131128/F036072.dat&hit=6) | | | 9.44/44.9 | 5.76/73 | 62 | 31% | 14 | R.EWNIEMK.S | | 949.4798 |  |
|  |  | | | | |  | | |  | | |  |  |  |  |  | R.GLVKYDSLTK.S | | 1123.4866 |  |
|  |  | | | | |  | | |  | | |  |  |  |  |  | K.YIADASSPNVGVTK.C | | 1421.6967 |  |
|  |  | | | | |  | | |  | | |  |  |  |  |  | K.RENDTMIQDIWR.L | | 1576.7585 |  |
|  |  | | | | |  | | |  | | |  |  |  |  |  | K.SIFHLAFVKATDYK.L | | 1639.9387 |  |
|  |  | | | | |  | | |  | | |  |  |  |  |  | K.SIFHLAFVKATDYK.L | | 1639.9387 |  |
|  |  | | | | |  | | |  | | |  |  |  |  |  | K.DLVEPSTPVAVCKNK.K | | 1656.7570 |  |
|  |  | | | | |  | | |  | | |  |  |  |  |  | R.VNAWRYLACTPSHR.I | | 1730.6968 |  |
|  |  | | | | |  | | |  | | |  |  |  |  |  | K.ISESCDGLFCIHSPK.S | | 1749.6729 |  |
|  |  | | | | |  | | |  | | |  |  |  |  |  | K.ISESCDGLFCIHSPK.S | | 1749.6729 |  |
|  |  | | | | |  | | |  | | |  |  |  |  |  | M.MEAEQQVVMASSSSLR.K | | 1752.6781 |  |
|  |  | | | | |  | | |  | | |  |  |  |  |  | M.MEAEQQVVMASSSSLRK.R | | 1880.9229 |  |
|  |  | | | | |  | | |  | | |  |  |  |  |  | M.MEAEQQVVMASSSSLRK.R | | 1880.9229 |  |
|  |  | | | | |  | | |  | | |  |  |  |  |  | R.WLRLLPPAGFQILIHK.F | | 1901.8716 |  |
|    \| **Energy and Metabolism** \| \| --- \| | | | | | | | | | | | | | | | | | | | | |
| 63 | transketolase, chloroplastic-like | | | | | *Glycine max* | | | [gi\|356576867](http://www.matrixscience.com/cgi/protein_view.pl?file=..%2Fdata%2F20131126%2FFTnorrceE.dat&hit=gi%7C356576867&db_idx=1&px=1&ave_thresh=47&_ignoreionsscorebelow=0&report=0&_sigthreshold=0.05&_msresflags=1025&_msresflags2=2&percolate=-1&percolate_rt=0&_minpeplen=7&sessionID=guest_guestsession) | | | 6.03/80.5 | 6.12/75 | 141 | 4% | 3 | K.NPTWFNR.D | | 934.4712 |  |
|  |  | | | | |  | | |  | | |  |  |  |  |  | R.FEALGWHVIWVK.N | | 1484.8087 |  |
|  |  | | | | |  | | |  | | |  |  |  |  |  | K.ALPTYTPESPADATR.N | | 1589.8038 |  |
| 77 | DEAD-box ATP-dependent RNA helicase | | | | | Japanese rice | | | [gi\|75328082](http://gps/mascot/cgi/protein_view.pl?file=../data/20131225/F038253.dat&hit=6) | | | 8.83/67.0 | 5.68/45 | 44 | 23% | 16 | K.GVNLLVATPGR.L | | 1096.5260 |  |
|  |  | | | | |  | | |  | | |  |  |  |  |  | K.DVMGAAKTGSGK.T | | 1121.4188 |  |
|  |  | | | | |  | | |  | | |  |  |  |  |  | R.HGISAANPYGR.K | | 1142.5935 |  |
|  |  | | | | |  | | |  | | |  |  |  |  |  | R.TTTFFNFCK.A | | 1165.5278 |  |
|  |  | | | | |  | | |  | | |  |  |  |  |  | -.MAPAPATTSSSKR.S + Oxidation (M) | | 1320.5380 |  |
|  |  | | | | |  | | |  | | |  |  |  |  |  | R.LLEQNFEEDMK.Q + Oxidation (M) | | 1411.7273 |  |
|  |  | | | | |  | | |  | | |  |  |  |  |  | R.TTTFFNFCKAEK.G | | 1493.6669 |  |
|  |  | | | | |  | | |  | | |  |  |  |  |  | K.KVMVFFSSCNSVK.F | | 1532.7023 |  |
|  |  | | | | |  | | |  | | |  |  |  |  |  | R.ELAIQTHNVAKELMK.Y | | 1724.7574 |  |
|  |  | | | | |  | | |  | | |  |  |  |  |  | R.ELAIQTHNVAKELMK.Y | | 1724.7574 |  |
|  |  | | | | |  | | |  | | |  |  |  |  |  | R.ELAIQTHNVAKELMK.Y + Oxidation (M) | | 1740.7489 |  |
|  |  | | | | |  | | |  | | |  |  |  |  |  | R.ELAIQTHNVAKELMK.Y + Oxidation (M) | | 1740.7489 |  |
|  |  | | | | |  | | |  | | |  |  |  |  |  | R.LLEQNFEEDMKQIFK.R | | 1911.7120 |  |
|  |  | | | | |  | | |  | | |  |  |  |  |  | R.LLEQNFEEDMKQIFK.R + Oxidation (M) | | 1927.7037 |  |
|  |  | | | | |  | | |  | | |  |  |  |  |  | K.ELMKYHSQTLGYIIGGNGR.R + Oxidation (M) | | 2152.9688 |  |
| 157 | Methionine adenosyltransferase | | | | | Catharanthus roseus | | | [gi\|3024127](http://www.matrixscience.com/cgi/protein_view.pl?file=..%2Fdata%2F20131126%2FFTnorrceS.dat&hit=gi%7C3024127&db_idx=1&px=1&ave_thresh=46&_ignoreionsscorebelow=0&report=0&_sigthreshold=0.05&_msresflags=1025&_msresflags2=2&percolate=-1&percolate_rt=0&_minpeplen=7&sessionID=guest_guestsession) | | | 5.51/43.4 | 6.06/44 | 162 | 18% | 5 | K.TAAYGHFGR.D | | 979.5360 |    \|  \| \| --- \| |
|  |  | | | | |  | | |  | | |  |  |  |  |  | R.TIFHLNPSGR.F | | 1141.6710 |  |
|  |  | | | | |  | | |  | | |  |  |  |  |  | R.FVIGGPHGDAGLTGR.K | | 1453.8276 |  |
|  |  | | | | |  | | |  | | |  |  |  |  |  | K.ENFDFRPGMIAINLDLK.R + Oxidation (M) | | 2009.1025 |  |
|  |  | | | | |  | | |  | | |  |  |  |  |  | K.VLVNIEQQSPDIAQGVHGHLTK.R | | 2383.3723 |  |
| 159 | enolase | | | | | Arabidopsis lyrata subsp. lyrata | | | [gi\|297323733](http://gps/mascot/cgi/protein_view.pl?file=../data/20131225/F038256.dat&hit=1) | | | 5.57/48 | 5.92/47 | 119 | 23% | 18 | K.KYDLNFK.E | | 927.4547 |    \|  \| \| --- \| |
|  |  | | | | |  | | |  | | |  |  |  |  |  | K.ARQIFDSR.G | | 992.4993 |  |
|  |  | | | | |  | | |  | | |  |  |  |  |  | R.AGWGVMASHR.S | | 1071.4719 |  |
|  |  | | | | |  | | |  | | |  |  |  |  |  | R.AGWGVMASHR.S | | 1071.4719 |  |
|  |  | | | | |  | | |  | | |  |  |  |  |  | R.AGWGVMASHR.S + Oxidation (M) | | 1087.4659 |  |
|  |  | | | | |  | | |  | | |  |  |  |  |  | K.MGVEVYHNLK.S | | 1189.5544 |  |
|  |  | | | | |  | | |  | | |  |  |  |  |  | K.MGVEVYHNLK.S + Oxidation (M) | | 1205.5447 |  |
|  |  | | | | |  | | |  | | |  |  |  |  |  | K.LGANAILAVSLAVCK.A | | 1499.7947 |  |
|  |  | | | | |  | | |  | | |  |  |  |  |  | K.VNQIGSVTESIEAVK.M | | 1573.7723 |  |
|  |  | | | | |  | | |  | | |  |  |  |  |  | R.IEEELGADAIYAGANFR.K | | 1838.8190 |  |
|  |  | | | | |  | | |  | | |  |  |  |  |  | R.IEEELGADAIYAGANFR.K | | 1838.8190 |  |
|  |  | | | | |  | | |  | | |  |  |  |  |  | K.LAMQEFMILPVGASSFK.E | | 1868.8757 |  |
|  |  | | | | |  | | |  | | |  |  |  |  |  | K.LAMQEFMILPVGASSFK.E | | 1868.8757 |  |
|  |  | | | | |  | | |  | | |  |  |  |  |  | K.LAMQEFMILPVGASSFK.E + Oxidation (M) | | 1884.8655 |  |
|  |  | | | | |  | | |  | | |  |  |  |  |  | K.LAMQEFMILPVGASSFK.E + 2 Oxidation (M) | | 1900.8508 |  |
|  |  | | | | |  | | |  | | |  |  |  |  |  | K.LAMQEFMILPVGASSFK.E + 2 Oxidation (M) | | 1900.8508 |  |
|  |  | | | | |  | | |  | | |  |  |  |  |  | K.LAMQEFMILPVGASSFKEAMK.M | | 2328.1042 |  |
|  |  | | | | |  | | |  | | |  |  |  |  |  | K.LAMQEFMILPVGASSFKEAMK.M + Oxidation (M) | | 2344.0911 |  |
| 181 | glutamine synthetase plant, putative | | | | | Ricinus communis | | | [gi\|223533499](http://gps/mascot/cgi/protein_view.pl?file=../data/20131225/F038257.dat&hit=1) | | | 6.11/39.3 | 5.46/44 | 64 | 16% | 6 | R.HKEHIAAYGK.G | | 1153.5538 |  |
|  |  | | | | |  | | |  | | |  |  |  |  |  | R.HKEHIAAYGK.G | | 1153.5538 |  |
|  |  | | | | |  | | |  | | |  |  |  |  |  | R.TLPGPVSDPSKLPK.W | | 1435.7229 |  |
|  |  | | | | |  | | |  | | |  |  |  |  |  | R.HETADINTFSWGVANR.G | | 1817.8063 |  |
|  |  | | | | |  | | |  | | |  |  |  |  |  | M.SLLADLININLSDSTDK.I | | 1831.7874 |  |
|  |  | | | | |  | | |  | | |  |  |  |  |  | M.SLLADLININLSDSTDK.I | | 1831.7874 |  |
| 194 | malate dehydrogenase | | | | | Lupinus albus | | | [gi\|27462762](http://www.matrixscience.com/cgi/protein_view.pl?file=..%2Fdata%2F20131126%2FFTnorrcmL.dat&hit=gi%7C27462762&db_idx=1&px=1&ave_thresh=46&_ignoreionsscorebelow=0&report=0&_sigthreshold=0.05&_msresflags=1025&_msresflags2=2&percolate=-1&percolate_rt=0&_minpeplen=7&sessionID=guest_guestsession) | | | 6.10/35.9 | 6.64/44 | 162 | 18% | 3 | K.LSSALSAASAACDHIR.D | | 1629.8545 |    \|  \| \| --- \| |
|  |  | | | | |  | | |  | | |  |  |  |  |  | R.ELVADDAWLNSEFIATVQQR.G | | 2305.2078 |  |
|  |  | | | | |  | | |  | | |  |  |  |  |  | K.GVVATTDVVEACTGVNIAVMVGGFPR.K | | 2619.3921 |  |
| 262 | proteasome subunit alpha type, putative | | | | | Ricinus communis | | | [gi\|223547282](http://gps/mascot/cgi/protein_view.pl?file=../data/20131225/F038262.dat&hit=1) | | | 5.73/25.5 | 5.50/44 | 248 | 41% | 17 | K.QAEQYHR.L | | 931.3975 |    \|  \| \| --- \| |
|  |  | | | | |  | | |  | | |  |  |  |  |  | K.EGFEGQISGK.N | | 1051.4517 |  |
|  |  | | | | |  | | |  | | |  |  |  |  |  | R.KQAEQYHR.L | | 1059.4836 |  |
|  |  | | | | |  | | |  | | |  |  |  |  |  | R.KQAEQYHR.L | | 1059.4836 |  |
|  |  | | | | |  | | |  | | |  |  |  |  |  | K.NIEIGIIGADK.K | | 1142.5730 |  |
|  |  | | | | |  | | |  | | |  |  |  |  |  | K.EPIPVTQLVR.E | | 1151.6228 |  |
|  |  | | | | |  | | |  | | |  |  |  |  |  | K.EPIPVTQLVR.E | | 1151.6228 |  |
|  |  | | | | |  | | |  | | |  |  |  |  |  | K.NIEIGIIGADKK.F | | 1270.6721 |  |
|  |  | | | | |  | | |  | | |  |  |  |  |  | K.KLPSILVDEASVQK.I | | 1526.8016 |  |
|  |  | | | | |  | | |  | | |  |  |  |  |  | R.LYKEPIPVTQLVR.E | | 1555.8450 |  |
|  |  | | | | |  | | |  | | |  |  |  |  |  | R.LYKEPIPVTQLVR.E | | 1555.8450 |  |
|  |  | | | | |  | | |  | | |  |  |  |  |  | K.LVQIEHALTAVGSGQTSLGIK.A | | 2122.0750 |  |
|  |  | | | | |  | | |  | | |  |  |  |  |  | K.LVQIEHALTAVGSGQTSLGIK.A | | 2122.0750 |  |
|  |  | | | | |  | | |  | | |  |  |  |  |  | R.YTDDMELDDAVHTAILTLK.E | | 2163.9302 |  |
|  |  | | | | |  | | |  | | |  |  |  |  |  | R.YTDDMELDDAVHTAILTLK.E + Oxidation (M) | | 2179.9895 |  |
|  |  | | | | |  | | |  | | |  |  |  |  |  | K.RYTDDMELDDAVHTAILTLK.E | | 2320.0110 |  |
|  |  | | | | |  | | |  | | |  |  |  |  |  | K.RYTDDMELDDAVHTAILTLK.E + Oxidation (M) | | 2336.0247 |  |
| 268 | glutathione s-transferase, putative | | | | | Ricinus communis | | | gi\|223527414 | | | 5.76/75.3 | 6.37/44 | 68 | 13% | 12 | K.GEELEAATK.E | | 947.5877 |  |
|  |  | | | | |  | | |  | | |  |  |  |  |  | R.FWADFVDK.K | | 1027.4368 |  |
|  |  | | | | |  | | |  | | |  |  |  |  |  | R.FWADFVDK.K | | 1027.4368 |  |
|  |  | | | | |  | | |  | | |  |  |  |  |  | R.FWADFVDKK.F | | 1155.5176 |  |
|  |  | | | | |  | | |  | | |  |  |  |  |  | R.FWADFVDKK.F | | 1155.5176 |  |
|  |  | | | | |  | | |  | | |  |  |  |  |  | K.FGNFSIEPECSK.L | | 1414.6534 |  |
|  |  | | | | |  | | |  | | |  |  |  |  |  | K.EDFSNKSALLLQSNPIHK.K | | 2040.8690 |  |
|  |  | | | | |  | | |  | | |  |  |  |  |  | K.LNVMLLCAMFQLYDAQRK.V + Oxidation (M) | | 2230.0154 |  |
|  |  | | | | |  | | |  | | |  |  |  |  |  | -.MANEVILLDFWPSMFGMRTR.I | | 2414.1074 |  |
|  |  | | | | |  | | |  | | |  |  |  |  |  | -.MANEVILLDFWPSMFGMRTR.I | | 2414.1074 |  |
|  |  | | | | |  | | |  | | |  |  |  |  |  | -.MANEVILLDFWPSMFGMRTR.I + Oxidation (M) | | 2430.1025 |  |
|  |  | | | | |  | | |  | | |  |  |  |  |  | -.MANEVILLDFWPSMFGMRTR.I + 2 Oxidation (M) | | 2446.0876 |  |
| 281 | Lon-related ATP-dependent protease, putative | | | | | *Medicago truncatula* | | | gi\|357441879 | | | 5.57 / 108.5 | 5.68/74 | 44 | 30% | 8 | K.HAARVMIPAR.Q | | 1121.4817 |  |
|  |  | | | | |  | | |  | | |  |  |  |  |  | K.LFEEEEDGR.S | | 1123.4919 |  |
|  |  | | | | |  | | |  | | |  |  |  |  |  | -.MATNLLLPPAAR.R + Oxidation (M) | | 1283.7113 |  |
|  |  | | | | |  | | |  | | |  |  |  |  |  | -.MATNLLLPPAARR.L + Oxidation (M) | | 1439.8129 |  |
|  |  | | | | |  | | |  | | |  |  |  |  |  | -.MATNLLLPPAARR.L + Oxidation (M) | | 1439.8129 |  |
|  |  | | | | |  | | |  | | |  |  |  |  |  | R.VNYLSPYFIETPLSMK.L | | 1901.8750 |  |
|  |  | | | | |  | | |  | | |  |  |  |  |  | R.KPSIVDNDLADVERALR.V | | 1910.8506 |  |
|  |  | | | | |  | | |  | | |  |  |  |  |  | K.VALITGAATSIGECIARSFCK.H | | 2225.1055 |  |
| 284 | tasselseed2-like short-chain dehydrogenase/reductase | | | | | *Melica altissima* | | | [gi\|88175017](http://gps/mascot/cgi/protein_view.pl?file=../data/20131128/F036074.dat&hit=2) | | | 5.99/26.3 | 6.01/74 | 45 | 28% | 6 | R.LFVRHGAK.V | | 927.5032 |  |
|  |  | | | | |  | | |  | | |  |  |  |  |  | R.LFVRHGAK.V | | 927.5032 |  |
|  |  | | | | |  | | |  | | |  |  |  |  |  | R.SLATLKGSTLRPR.D | | 1399.6912 |  |
|  |  | | | | |  | | |  | | |  |  |  |  |  | R.VNALGAALGMKHAAR.A | | 1479.7992 |  |
|  |  | | | | |  | | |  | | |  |  |  |  |  | R.VNALGAALGMKHAAR.A | | 1479.7992 |  |
|  |  | | | | |  | | |  | | |  |  |  |  |  | K.SILSFDAGEFDRVLR.V | | 1724.8397 |  |
|  |  | | | | |  | | |  | | |  |  |  |  |  | K.SILSFDAGEFDRVLR.V | | 1724.8397 |  |
|  |  | | | | |  | | |  | | |  |  |  |  |  | R.GIRVNCVSPFGVATPMLINAWR.Q | | 2458.1628 |  |
| 373 | pyruvate dehydrogenase, putative | | | | | *Ricinus communis* | | | [gi\|223548594](http://gps/mascot/cgi/protein_view.pl?file=../data/20131128/F036085.dat&hit=1) | | | 5.95/39.7 | 5.23/36 | 83 | 21% | 12 | K.GLLDKYGPER.V | | 1147.5944 |  |
|  |  | | | | |  | | |  | | |  |  |  |  |  | K.EGISAEVINLR.S | | 1200.6593 |  |
|  |  | | | | |  | | |  | | |  |  |  |  |  | K.EGISAEVINLR.S | | 1200.6593 |  |
|  |  | | | | |  | | |  | | |  |  |  |  |  | R.MAVPQVEDIVR.A | | 1256.6630 |  |
|  |  | | | | |  | | |  | | |  |  |  |  |  | R.MAVPQVEDIVR.A | | 1256.6630 |  |
|  |  | | | | |  | | |  | | |  |  |  |  |  | R.MAVPQVEDIVR.A + Oxidation (M) | | 1272.6530 |  |
|  |  | | | | |  | | |  | | |  |  |  |  |  | R.MAVPQVEDIVR.A + Oxidation (M) | | 1272.6530 |  |
|  |  | | | | |  | | |  | | |  |  |  |  |  | R.IAGADVPMPYAANLER.M + Oxidation (M) | | 1703.8307 |  |
|  |  | | | | |  | | |  | | |  |  |  |  |  | K.STYMSAGQLSVPIVFR.G | | 1755.8710 |  |
|  |  | | | | |  | | |  | | |  |  |  |  |  | K.STYMSAGQLSVPIVFR.G | | 1755.8710 |  |
|  |  | | | | |  | | |  | | |  |  |  |  |  | K.STYMSAGQLSVPIVFR.G + Oxidation (M) | | 1771.8854 |  |
|  |  | | | | |  | | |  | | |  |  |  |  |  | K.VFLMGEEVGEYQGAYK.I + Oxidation (M) | | 1835.8346 |  |
| 402 | pyruvate dehydrogenase, putative | | | | | *Ricinus communis* | | | [gi\|223548594](http://gps/mascot/cgi/protein_view.pl?file=../data/20131128/F036085.dat&hit=1) | | | 5.95/39.7 | 6.17/28 | 83 | 21% | 12 | K.GLLDKYGPER.V | | 1147.5944 |  |
|  |  | | | | |  | | |  | | |  |  |  |  |  | K.EGISAEVINLR.S | | 1200.6593 |  |
|  |  | | | | |  | | |  | | |  |  |  |  |  | K.EGISAEVINLR.S | | 1200.6593 |  |
|  |  | | | | |  | | |  | | |  |  |  |  |  | R.MAVPQVEDIVR.A | | 1256.6630 |  |
|  |  | | | | |  | | |  | | |  |  |  |  |  | R.MAVPQVEDIVR.A | | 1256.6630 |  |
|  |  | | | | |  | | |  | | |  |  |  |  |  | R.MAVPQVEDIVR.A + Oxidation (M) | | 1272.6530 |  |
|  |  | | | | |  | | |  | | |  |  |  |  |  | R.MAVPQVEDIVR.A + Oxidation (M) | | 1272.6530 |  |
|  |  | | | | |  | | |  | | |  |  |  |  |  | R.IAGADVPMPYAANLER.M + Oxidation (M) | | 1703.8307 |  |
|  |  | | | | |  | | |  | | |  |  |  |  |  | K.STYMSAGQLSVPIVFR.G | | 1755.8710 |  |
|  |  | | | | |  | | |  | | |  |  |  |  |  | K.STYMSAGQLSVPIVFR.G | | 1755.8710 |  |
|  |  | | | | |  | | |  | | |  |  |  |  |  | K.STYMSAGQLSVPIVFR.G + Oxidation (M) | | 1771.8854 |  |
|  |  | | | | |  | | |  | | |  |  |  |  |  | K.VFLMGEEVGEYQGAYK.I + Oxidation (M) | | 1835.8346 |  |
| **Redox Homeostasis** | | | | | | | | | | | | | | | | | | | | |
| 336 | Thioredoxin H-type, putative | | | | | *Ricinus communis* | | | gi\|255555063 | | | 4.70/13.8 | 5.87/44 | 24 | 2% | 1 | AMPTFVLMKNGAQVDK | | 1749.6412 |  |
| **Unknown** | | | | | | | | | | | | | | | | | | | | |
| 54 | hypothetical protein | | | | | Arabidopsis lyrata subsp. lyrata | | | [gi\|297328147](http://gps/mascot/cgi/protein_view.pl?file=../data/20131225/F038252.dat&hit=1) | | | 6.10/85.37 | 5.50/90 | 116 | 20% | 15 | K.SWLAFAAQK.V | | 1021.4892 |  |
|  |  | | | | |  | | |  | | |  |  |  |  |  | K.YLFAGVVDGR.N | | 1096.5222 |  |
|  |  | | | | |  | | |  | | |  |  |  |  |  | K.YLFAGVVDGR.N | | 1096.5222 |  |
|  |  | | | | |  | | |  | | |  |  |  |  |  | R.SDEKLLSVFR.E | | 1193.5588 |  |
|  |  | | | | |  | | |  | | |  |  |  |  |  | K.FALESFWDGK.S | | 1199.5128 |  |
|  |  | | | | |  | | |  | | |  |  |  |  |  | K.GGIGVIQIDEAALR.E | | 1411.7236 |  |
|  |  | | | | |  | | |  | | |  |  |  |  |  | K.GGIGVIQIDEAALR.E | | 1411.7236 |  |
|  |  | | | | |  | | |  | | |  |  |  |  |  | M.ASHIVGYPRMGPK.R + Oxidation (M) | | 1428.6757 |  |
|  |  | | | | |  | | |  | | |  |  |  |  |  | R.SDIWKQMSAAGIK.Y | | 1434.7015 |  |
|  |  | | | | |  | | |  | | |  |  |  |  |  | K.YGAGIGPGVYDIHSPR.I | | 1658.7524 |  |
|  |  | | | | |  | | |  | | |  |  |  |  |  | K.YGAGIGPGVYDIHSPR.I | | 1658.7524 |  |
|  |  | | | | |  | | |  | | |  |  |  |  |  | K.GMLTGPVTILNWSFVR.N | | 1790.8792 |  |
|  |  | | | | |  | | |  | | |  |  |  |  |  | K.GMLTGPVTILNWSFVR.N | | 1790.8792 |  |
|  |  | | | | |  | | |  | | |  |  |  |  |  | K.GMLTGPVTILNWSFVR.N + Oxidation (M) | | 1806.8682 |  |
|  |  | | | | |  | | |  | | |  |  |  |  |  | K.LVVSTSCSLLHTAVDLVNETK.L | | 2286.0803 |  |
|  |  | | | | |  | | |  | | |  |  |  |  |  | K.LNLPILPTTTIGSFPQTVELR.R | | 2310.1965 |  |
|  |  | | | | |  | | |  | | |  |  |  |  |  | K.LNLPILPTTTIGSFPQTVELR.R | | 2310.1965 |  |
|  |  | | | | |  | | |  | | |  |  |  |  |  | K.KLNLPILPTTTIGSFPQTVELR.R | | 2438.2856 |  |
| 155 | hypothetical protein | | | | | Oryza sativa Indica Group | | | [gi\|125556971](http://gps/mascot/cgi/protein_view.pl?file=../data/20131225/F038255.dat&hit=1) | | | 8.01/54.0 | 5.98/46 | 150 | 21% | 14 | K.GILESLRR.R | | 943.5211 |  |
|  |  | | | | |  | | |  | | |  |  |  |  |  | K.EVAEFIER.R | | 992.4919 |  |
|  |  | | | | |  | | |  | | |  |  |  |  |  | K.EVAEFIER.R | | 992.4919 |  |
|  |  | | | | |  | | |  | | |  |  |  |  |  | K.GYWGECGQR.G | | 1112.4912 |  |
|  |  | | | | |  | | |  | | |  |  |  |  |  | R.KEVAEFIER.R | | 1120.5795 |  |
|  |  | | | | |  | | |  | | |  |  |  |  |  | K.VLLDMGPPISK.E | | 1169.6008 |  |
|  |  | | | | |  | | |  | | |  |  |  |  |  | R.IMTDGFNSCR.N | | 1200.4583 |  |
|  |  | | | | |  | | |  | | |  |  |  |  |  | R.IMTDGFNSCR.N | | 1200.4583 |  |
|  |  | | | | |  | | |  | | |  |  |  |  |  | R.IMTDGFNSCR.N + Oxidation (M) | | 1216.4517 |  |
|  |  | | | | |  | | |  | | |  |  |  |  |  | R.KVLLDMGPPISK.E | | 1297.6486 |  |
|  |  | | | | |  | | |  | | |  |  |  |  |  | K.EVQLVSFHTVSK.G | | 1373.6744 |  |
| 193 | unknown | | | | | *Picea sitchensis* | | | gi\|294462854 | | | 5.3/38.3 | 5.59/44 | 42 | 5% | 1 | MLLQNVPPALLVR + Oxidation (M) | | 1479.8234 |  |
| 224 | uncharacterized protein | | | | | *Glycine max* | | | [gi\|359806184](http://www.matrixscience.com/cgi/protein_view.pl?file=..%2Fdata%2F20131126%2FFTnorrcem.dat&hit=gi%7C359806184&db_idx=1&px=1&ave_thresh=45&_ignoreionsscorebelow=0&report=0&_sigthreshold=0.05&_msresflags=1025&_msresflags2=2&percolate=-1&percolate_rt=0&_minpeplen=7&sessionID=guest_guestsession) | | | 6.21/29.9 | 4.71/44 | 61 | 5% | 1 | K.LFVGNLPFSVDSAR.L | | 1521.8612 |  |
| 337 | unknown | | | | | *Glycine max* | | | gi\|255627871 | | | 6.36/16.9 | 6.00/44 | 31 | 5% | 1 | LDKVYKPAPVVLINR | | 1724.8145 |  |
| 341 | predicted protein | | | | | *Micromonas* | | | gi\|255089250 | | | 10.5/47.5 | 5.72/43 | 37 | 3% | 1 | AMGANSRPTPAHR | | 1365.6451 |  |
| 398**^** | unnamed protein product | | | | | *Vitis vinifera* | | | gi\|296086099 | | | 5.95/42.8 | 5.05/37 | 114 | 13% | 8 | K.LFSPGNLR.A | | 903.4614 |    \|  \| \| --- \| |
|  |  | | | | |  | | |  | | |  |  |  |  |  | K.LFSPGNLR.A | | 903.4614 |  |
|  |  | | | | |  | | |  | | |  |  |  |  |  | K.LINYYVR.E | | 940.4675 |  |
|  |  | | | | |  | | |  | | |  |  |  |  |  | R.FEETLYGSSR.L | | 1188.4979 |  |
|  |  | | | | |  | | |  | | |  |  |  |  |  | R.FEETLYGSSR.L | | 1188.4979 |  |
|  |  | | | | |  | | |  | | |  |  |  |  |  | R.LLFEVAPLGFLIEK.A | | 1588.8458 |  |
|  |  | | | | |  | | |  | | |  |  |  |  |  | R.LLFEVAPLGFLIEK.A | | 1588.8458 |  |
|  |  | | | | |  | | |  | | |  |  |  |  |  | R.YTGGMVPDVNQIIVK.E + Oxidation (M) | | 1649.7623 |  |
| 485 | unnamed protein product | | | | | Vitis vinifera | | | [gi\|297744970](http://gps/mascot/cgi/protein_view.pl?file=../data/20131225/F038272.dat&hit=1) | | | 4.79/13.4 | 6.24/14 | 69 | 37% | 7 | R.DDFVEKDR.S | | 1023.4164 |  |
|  |  | | | | |  | | |  | | |  |  |  |  |  | R.EITLGFVDLLR.D | | 1275.6553 |  |
|  |  | | | | |  | | |  | | |  |  |  |  |  | R.EITLGFVDLLR.D | | 1275.6553 |  |
|  |  | | | | |  | | |  | | |  |  |  |  |  | K.EIKFEFPAMDTL.- | | 1440.6241 |  |
|  |  | | | | |  | | |  | | |  |  |  |  |  | K.EIKFEFPAMDTL.- + Oxidation (M) | | 1456.6141 |  |
|  |  | | | | |  | | |  | | |  |  |  |  |  | K.WSPELAAACEVWK.E | | 1546.6465 |  |
|  |  | | | | |  | | |  | | |  |  |  |  |  | R.EITLGFVDLLRDDFVEK.D | | 2008.9258 |  |

**Supplemental Table S2: The primer sequences for real time PCR**

| Spot No. | Gene name | Forward (5’-3’) | Reverse (5’-3’) | Tm (°C) | Cycle number | Product length (bp) |
| --- | --- | --- | --- | --- | --- | --- |
|  | *ACTIN* | TCCATAATGAAATGTGATGTTG  TTAATGCTACAAAGGCAGCT | GGAAGGTGCTTAGAGATGC  GCAACATCTGACCACCTTT | 53 | 30 | 217 |
| 99 | *RULSB* |  |  | 52 | 35 | 318 |
| 446 | *RBCL* | CTGATATCTTGGCAGCATTC | AATTTATCTCTCTCAACTTGGA | 52 | 30 | 385 |
| 308 | *ASCF1* | TGTCTCTTCTATTACGAAGACC | CAGCTACTTGTTTCATGGCT | 54 | 35 | 321 |
| 422 | *LETN* | GTTGCAGTTGAATTTGACAC | GAAACCAACTCTTACCCACT | 51 | 40 | 267 |
| 423 | *FTN* | TATCAGAACATTCGTGGTGG | TGACCCTTTCCAACTAATCTC | 56 | 35 | 284 |
| 281 | *LADP* | TTGTTGGCAAGCCTGTCT | ACTTCCCCAGTCATTGCTA | 54 | 35 | 397 |
| 284 | *TS2* | ATGCTGCAAGGGTGATGAT | CCAAATGGTGAAATGCAATT | 50 | 40 | 190 |
| 63 | *TRKT* | AGCCATTAAGGAAGCAAAG | TCTTCTGGCACATGGAAAG | 53 | 35 | 189 |
| 413 | *APX* | GTTTGGCATGGCACTCTG | GTGGTGGCTCAGGCTTGT | 56 | 35 | 267 |
| 280 | *CHL2* | CTCTCTTGATTGCCACTCCT | TTTCCTTTGTCCATTCCATC | 51 | 40 | 397 |
| 213 | *FNR* | ATGGTTTGGCATGGCTCT | TCATCAATTCCCTTTTCCATT | 52 | 35 | 265 |
| 47 | *HSP* | AGGAGGTGTCTCATGAGTGG | GGCTTCTTCCTTGTGTCAAA | 55 | 40 | 232 |
| 155 | *ATT* | CTTAGTCCAAATGTTCCAGCA | TCTGTCCAAATCCTGAACCA | 55 | 40 | 337 |
| 366 | *FBA2* | CCAGAGATCTTGCTTGATGG | GACACGTGCCATGGGTTT | 55 | 40 | 326 |
| 159 | *ENO2* | GTCATTGGAATGGATGTTGC | ATAGTGCTCCCAGTCATCCT | 55 | 40 | 198 |
| 262 | *PSA* | TTTTCTTGGAAAGCTTCTGC | GGTGTCAATACCCTGAACTT | 53 | 40 | 206 |
| 256 | *TIM* | GGGTTCGCAAAGGTGGT | GGCCCAAACTGGCTCATA | 55 | 40 | 308 |
| 157  45 | *MAT2* | GGTTGATAGGAGTGGTGCTTA | CCACTTCCCATGTGAAGTC | 55 | 40 | 317 |
|  | *ClPC* | TGGTGTGGGGAAGTCTGA | TCAATCACACTGCTTCCAAC | 55 | 40 | 357 |
| 279 | *LR10* | TTGCCATGGCAATTGCT | CTAGAGTAGTTTCAAGCATGTCT | 51 | 40 | 281 |
